# Effects of post-harvest ‘ rubbing-sweating ‘ drying treatment on the accumulation of bioactive compounds in *Codonopsis pilosula*: a transcriptomic analysis

**DOI:** 10.3389/fpls.2025.1650787

**Published:** 2025-09-30

**Authors:** Wei Liang, Gang Bai, Jiachen Sun, Wenzhen Tao, Qian Li, Pengbin Dong, Hongyan Wang, Jiali Cheng, Fengxia Guo, Yuan Chen

**Affiliations:** ^1^ State Key Laboratory of Arid Land Crop Science, College of Agronomy, College of Life Science and Technology, Gansu Agricultural University, Lanzhou, China; ^2^ School of Biotechnology and Food Science, Tianjin University of Commerce, Tianjin, China; ^3^ Jingyuan Road Community Health Center Chengguan District, Lanzhou, China

**Keywords:** Codonopsis Radix, rubbing-sweating dried, shade-dried, postharvest processing, stress response genes, polysaccharide and lobetyolin biosynthesis

## Abstract

Postharvest processing critically determines the quality of *Codonopsis pilosula*, yet the molecular mechanisms underlying the traditional “rubbing–sweating” technique remain unclear. We hypothesized that rubbing–sweating imposes stronger abiotic stress than shade drying, thereby activating stress-responsive pathways and enhancing the accumulation of bioactive constituents. To test this, freshly harvested roots were processed by shade drying (SD) and rubbing–sweating drying (RD), and compared with fresh controls (FC) in terms of chemical composition, antioxidant enzyme activity, and transcriptomic profiles. After 6 days, RD significantly increased lobetyolin content by 15.3% relative to FC and 9.7% relative to SD (*p*<0.01), while polysaccharides reached 19.5% in RD versus 10.6% in FC (*p*<0.05). Antioxidant enzymes also exhibited marked increases under RD, with catalase activity elevated by 235% compared to FC. Transcriptome sequencing revealed 17,338 DEGs in RD vs. SD and 11,007 in RD vs. FC, enriched in MAPK signaling, hormone transduction, and aromatic amino acid biosynthesis. These findings support our hypothesis that rubbing–sweating enhances the medicinal quality of *C. pilosula* through stress-induced activation of metabolic pathways. This work provides the first transcriptomic evidence for the molecular basis of this traditional technique, offering new insights for optimizing and modernizing postharvest processing.

## 1 Introduction

In recent years, rising living standards and advancements in the pharmaceutical industry have led to a growing annual demand for high-quality traditional Chinese medicinal (TCM) materials with health-promoting properties. The quality of TCM is determined by a complex interplay of factors, including the growing environment, cultivation practices, and duration of growth, as well as appropriate post-harvest processing methods at the production site, which are critical to the final quality of the medicinal materials (Li et al., 2020; Chen et al., 2023). Common traditional post-harvest processing methods for Chinese medicinal materials include steaming, boiling, blanching, smoking and “Rubbing-sweating” (Zhang et al., 2024; Xu et al., 2025). However, some of these methods are labor-intensive, time-consuming, and technically complex, rendering them increasingly incompatible with the modernization needs of the traditional Chinese medicine (TCM) industry. Therefore, it is essential to conduct in-depth investigations into traditional processing techniques to elucidate their underlying scientific mechanisms. Such research can offer valuable insights for developing processing methods that not only improve the quality of medicinal materials but also align with the demands of modern industrial production.

Codonopsis Radix (Dangshen) refers to the dried roots of perennial species in the Campanulaceae family, including *Codonopsis pilosula* (Franch.) Nannf., *C. pilosula* Nannf. var. *modesta* (Nannf.) L.T. Shen, and *C. tangshen* Oliv (Liang et al., 2024). Due to its functions in strengthening the spleen, moistening the lungs, nourishing the blood, promoting the generation of bodily fluids, modulating immune function, and exhibiting antitumor properties (Zou et al., 2014; Bai et al., 2018), *C. pilosula* has been used for centuries in both food and traditional folk medicine across Asian countries, including China, Japan, and Korea (Luan et al., 2021). In the traditional production areas of *C. pilosula*, a distinctive post-harvest processing technique known as “Rubbing-sweating” has gradually evolved through long-term practice. Locally, it is widely believed that *C. pilosula* processed using this method possesses a superior commercial appearance and enhanced quality. The traditional rubbing and sweating process consists of the following steps: (1) Freshly harvested roots are cleaned to remove impurities and shade-dried for 2–3 days until their texture changes from hard to pliable; (2) The roots are bundled and vigorously rubbed by hand or with mechanical assistance to make the tissues denser and firmer; (3) The rubbed roots are heaped and covered with straw mats to allow surface moisture to condense. This sweating step lasts 1–2 days, with careful monitoring to prevent mold or spoilage; (4) The roots are then sun-dried for 1–2 days. Steps (2) and (3) are typically repeated 2–3 times until the roots are fully dried (Liang et al., 2024).

Freshly harvested plant tissues—particularly roots and rhizomes—retain substantial physiological activity for a period following harvest. During this time, they can respond to external stimuli by initiating a series of physiological and biochemical reactions, thereby influencing metabolic processes and the accumulation of secondary metabolites within the plant (Qi et al., 2024). Studies have demonstrated that appropriate postharvest processing methods can significantly enhance the accumulation of volatile C6-compounds in oolong tea (Zhou et al., 2020). Similarly, postharvest high-temperature treatment of jujube fruit (soaking in 50°C water for 4 minutes) has been shown to effectively delay senescence and decay (Yang et al., 2021). In addition, the traditional “sweating” process has been employed in the postharvest handling of several Chinese medicinal materials, including *Magnolia officinalis*, *Salvia miltiorrhiza*, and *Gentiana macrophylla*. Some researchers have suggested that sweating alters the composition of microbial communities in *M. officinalis*, thereby increasing the content of active compounds such as magnolol and honokiol in the final product (Wu et al., 2019). Others have found that sweating significantly promotes the production of intermediate metabolites involved in the biosynthesis of tanshinones and salvianolic acids in *S. miltiorrhiza*, contributing to the accumulation of these bioactive components and improving the quality of the dried material (Cao et al., 2020). Furthermore, sweating has been reported to enhance the biosynthesis of phenylalanine, tyrosine, and tryptophan, as well as polyphenol and terpene biosynthesis in *G. macrophylla*, thus promoting the accumulation of key active ingredients and improving the overall quality of the herb (Sun et al., 2023).

At present, it remains unclear how the traditional “rubbing–sweating” process affects the active components of *C. pilosula* and its molecular regulatory mechanism. We hypothesized that the “rubbing–sweating” treatment might enhance the quality of *C. pilosula* by inducing the stress response signaling pathway and promoting the accumulation of bioactive compounds. To verify this hypothesis, this study compared the differences in chemical components, antioxidant enzyme activities, and transcriptome profiles among fresh *C. pilosula*, shade-dried *C. pilosula*, and *C. pilosula* treated with the “rubbing–sweating” process. The aim of this study was to clarify the molecular mechanism by which the “rubbing–sweating” treatment improves the quality of *C. pilosula*, providing a theoretical basis for the optimization of traditional processing techniques and the development of modern drying technologies.

## 2 Materials and methods

### 2.1 Plant material and experimental design

Root samples of *C. pilosula* were collected from Wen County, Gansu Province, China (32.944° N, 104.683° E), and were of uniform age (3 years). Botanical identification was performed by Professor Chen Yuan from the College of Agronomy, Gansu Agricultural University, who confirmed the samples as members of the Campanulaceae family, specifically *Codonopsis pilosula* (Franch.) Nannf.

The collected *C. pilosula* samples, selected for uniform size, were divided into three groups, each consisting of 12 roots. One group was designated as the control (fresh *C. pilosula*, FC), while the other two groups were subjected to shade-drying (SD) and “Rubbing-sweating” drying (RD) treatments, respectively. For subsequent analyses, three biological replicates were established per treatment, with each replicate comprising pooled tissues from four randomly selected roots.

#### 2.1.1 Fresh *C. pilosula*

The freshly harvested *C. pilosula* samples were immediately stored at −80°C to preserve them for subsequent analysis.

#### 2.1.2 “Rubbing-sweating” dried *C. pilosula*

The samples were kept indoors and manually rubbed every three days. After each rubbing session, the roots were covered with a breathable tarp for 12 hours to facilitate sweating, followed by air-drying for two days to promote moisture loss and prevent mold growth. This cycle was repeated three times. The ambient temperature was maintained at 2–10°C throughout the process.

#### 2.1.3 Shade-dried *C. pilosula*

The samples were placed in a cool, shaded environment under the same conditions as the RD group, except without rubbing or sweating. They were dried continuously for 12 days, with air-drying every three days to facilitate moisture loss and prevent mold formation.

To investigate transcriptomic changes during the drying process, samples were collected after four days of treatment. Each sample weighed 9 g and was immediately stored in an ultra-low-temperature freezer (−80°C) for further analysis. Voucher specimens of *C. pilosula* used in this study were deposited in the Herbarium of Gansu Agricultural University (College of Agronomy) to facilitate future research and verification.

### 2.2 Detection of chemical composition

Freeze-dried *C. pilosula* tissue (2 g) was ground into a fine powder and extracted with 20 mL of 50% ethanol using ultrasonic assistance at 60°C for 60 minutes. The extract was then centrifuged at 1000 rpm for 5 minutes, and the resulting supernatant was filtered through a 0.22 μm organic membrane filter. The filtrate was subsequently analyzed using a high-performance liquid chromatography with photodiode array detection (HPLC-PDA) system. Quantitative determination of five major chemical constituents—adenosine, protocatechuic acid, tryptophan, syringin, and lobetyolin—was performed using an Agilent 1260 liquid chromatograph equipped with a Kromasil C_18_ reversed-phase column (4.6 mm × 250 mm). The mobile phase consisted of 0.5% formic acid aqueous solution (solvent A) and acetonitrile (solvent B), delivered at a flow rate of 1 mL·min^-^¹. The gradient elution program was as follows: 0–5 min, 5% B; 5–10 min, 5–8% B; 10–15 min, 8% B; 15–25 min, 8–14% B; 25–30 min, 14–20% B; 30–40 min, 20–30% B; and 40–50 min, 30–50% B. The injection volume was 10 μL, and detection was carried out at 260 nm.

The contents of polysaccharides, proteins, and ash were determined in accordance with AOAC standard methods (Iyda et al., 2019; Yang et al., 2019).

### 2.3 Superoxide dismutase, peroxidase, catalase, and ascorbate peroxidase activity assay

A total of 1.00 g of fresh *C. pilosula* tissue powder was mixed with 9 mL of normal saline for enzymatic extraction. The mixture was centrifuged at 2,500 rpm for 10 minutes at 4°C, and the resulting supernatant was collected for the determination of superoxide dismutase (SOD), peroxidase (POD), catalase (CAT), and ascorbate peroxidase (APX) activities. Enzyme activity assay kits were purchased from Nanjing Jiancheng Bioengineering Institute.

### 2.4 Transcriptome data determination

#### 2.4.1 RNA abstraction and quality evaluation

Samples from FC, SD (6 days), and RD (6 days) groups (0.5 g fresh weight each) were selected for total RNA extraction using TRIzol reagent (Invitrogen, Carlsbad, CA, USA), following the manufacturer’s instructions. The integrity and concentration of the extracted RNA were assessed using a 2100 Bioanalyzer (Agilent Technologies, Santa Clara, CA, USA) and quantified using a NanoDrop ND-2000 spectrophotometer (Thermo Scientific, Wilmington, DE, USA). High-quality RNA was subsequently used for library construction.

#### 2.4.2 Complementary DNA library transcription group fabrication and sequencing

RNA purification, reverse transcription, library construction, and sequencing were performed by EMajorbio Bio-Pharm Biotechnology Co., Ltd. (Shanghai, China). RNA-seq libraries were prepared using the TruSeq™ RNA Sample Preparation Kit (Illumina, USA). Poly(A) mRNA was isolated from total RNA using oligo(dT)-attached magnetic beads, followed by fragmentation using a fragmentation buffer. The resulting short RNA fragments were used as templates to synthesize double-stranded complementary DNA (ds-cDNA) using the SuperScript Double-Stranded cDNA Synthesis Kit (Invitrogen) and RHP reagents (Illumina). Subsequently, the cDNA underwent end repair, phosphorylation, and addition of a single ‘A’ base according to Illumina’s library preparation protocol. Libraries were size-selected for cDNA fragments of 200–300 bp using 2% low-range ultra agarose (LRUA) gel and amplified via PCR using Phusion High-Fidelity DNA Polymerase for 15 cycles. After quantification using the TBS-380 fluorometer, the libraries were sequenced on an Illumina HiSeq X Ten or NovaSeq 6000 platform to generate 2 × 150 bp paired-end reads.

#### 2.4.3 *De novo* assembling and annotation

The original paired end reads were treated with trimming and QC by SeqPrep and Sickle. Afterwards, the cleaning data from the samples were adopted to perform *de novo* assembling with Trinity (Grabherr et al., 2011). The entire assembling transcription products were retrieved against the National Center for Biotechnology Information (NCBI) protein NR (https://www.ncbi.nlm.nih.gov/public/, July 2023), GO (http://www.geneontology.org/, July 2023), and KEGG (http://www.genome.jp/kegg/, September 2023) data centers via BLASTX (2.9.0) to determine the proteins with the greatest sequential resemblance compared to the aforementioned transcription products to search the functional notations and a representative cut-off E-values<1.0 × 10^−5^ was set. BLAST2GO software (Agu 2018) was adopted to acquire gene ontology (GO) notations of distinctive assembling transcription products for the description of biology activities, molecule roles and cell constituents. Metabolism pathway assay was completed via the KEGG method (Conesa et al., 2005).

#### 2.4.4 Differential expression analysis and functional enrichment

To identify differentially expressed genes (DEGs) between groups, transcript expression levels were calculated using the transcripts per million (TPM) method. Gene abundances were quantified using RSEM (Li and Dewey, 2011). Differential expression analysis was conducted using either DESeq2 (Love et al., 2014) or DEGseq (Wang et al., 2010). Genes with |log_2_ fold change| ≥ 1 and false discovery rate (FDR)<0.05 (DESeq2) or FDR<0.001 (DEGseq) were considered significantly differentially expressed. Furthermore, functional enrichment analyses, including Gene Ontology (GO) and Kyoto Encyclopedia of Genes and Genomes (KEGG) pathway analyses, were performed to identify significantly enriched GO terms and metabolic pathways. Enrichment was evaluated using a Bonferroni-corrected *p*-value<0.05 compared with the whole transcriptome background. GO and KEGG analyses were conducted using GOATOOLS (https://pypi.org/project/goatools/, Version 1.4.4) and the Python SciPy library (https://docs.scipy.org/doc/, Version 1.10.0), respectively.

### 2.5 Quantitative real-time PCR validation

Overall RNA was abstracted via Plant RNA Kit II (OMEGA, Norcross, USA). The first normal cDNA was prepared from overall RNA via a PrimeScript™ RT Reagent Kit with gDNA Eraser (Takara, Dalian, China). Actin was chosen to be an inner control. The heat cycle procedurefor quantitative reverse transcription polymerase chain reaction (qRT-PCR): incipient denaturating at 95°Cfor 2min, denaturating at 94°C for 30 s, annealing at 58°C for 30 s and elongation at 72°Cfor 38s, for an overall 40 cycles. The primers adopted for qRT-PCR herein were presented by Supporting Information (**Supplementary Table S2**). Each assay was completed three times. The comparative expression level of every unigene was calculated via the 2^−ΔΔCt^ approach.

### 2.6 Statistical analysis

All experiments were performed with three independent biological replicates per treatment, each consisting of pooled tissues from four roots, and with three technical replicates for each assay. Data are presented as mean±standard error (SE). Statistical analysis was performed using SPSS version 26.0 (SPSS Inc., Chicago, IL, USA). Duncan’s multiple range test was used to determine significant differences among groups, with different uppercase letters indicating significance at *p*<0.01 and different lowercase letters indicating significance at *p*<0.05. Graphs were generated using OriginPro 2021 (OriginLab Corporation, Northampton, MA, USA).

## 3 Results

### 3.1 Effect of dry method on the chemical and nutritional compositions of *C. pilosula*

**Figure 1:**
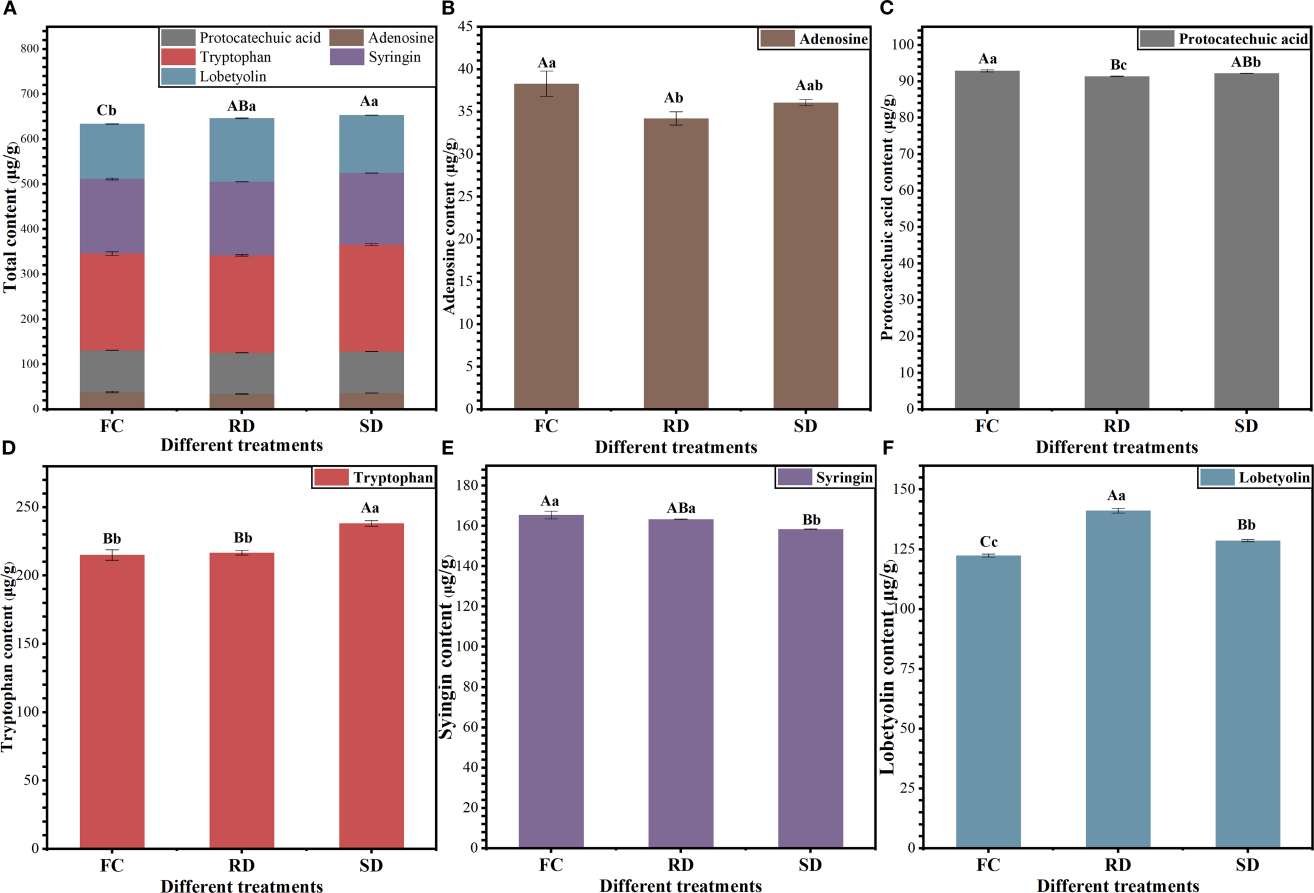
Five active ingredient analysis of Fresh (FC), “Rubbing-sweating” dry (RD) and Shade-dry (SD) *C. pilosula*. The error bars represent the means±SE (n=3). Different letters indicate a significant difference (capital letters *p*<0.01, small letters *p*<0.05).

As shown in **Figure 1**, the levels of active ingredients in *C. pilosula* changed significantly after a 6-day drying period (*p*<0.05). The total content of five major active components was significantly higher in the RD and SD samples compared to the FC samples, with increases of 2.01% and 3.06%, respectively (*p*<0.05; **Figure 1A**). The adenosine content in FC (38.30 μg/g) was significantly higher than in RD (34.21 μg/g; *p*<0.05), but not significantly different from SD (36.07 μg/g; *p* > 0.05; **Figure 1B**). Similarly, the protocatechuic acid content in FC (92.90 μg/g) was significantly higher than in RD (91.42 μg/g) and SD (92.19 μg/g) (*p*<0.05; **Figure 1C**). In contrast, tryptophan content in SD (238.22 μg/g) was significantly higher than in both FC (215.08 μg/g) and RD (216.75 μg/g) (*p*<0.05; **Figure 1D**). For syringin, the content in FC (165.45 μg/g) and RD (163.37 μg/g) was significantly higher than in SD (158.46 μg/g) (*p*<0.05; **Figure 1E**). Notably, lobetyolin content was highest in RD (141.18 μg/g), which was significantly higher than in FC (122.44 μg/g) and SD (128.67 μg/g) (*p*<0.01; **Figure 1F**), with increases of 15.31% and 9.72%, respectively. These findings suggest that different drying treatments exert distinct effects on the accumulation of active components in *C. pilosula*. Among them, the “Rubbing-sweating” method appears to be particularly effective in promoting lobetyolin accumulation.

**Figure 2:**
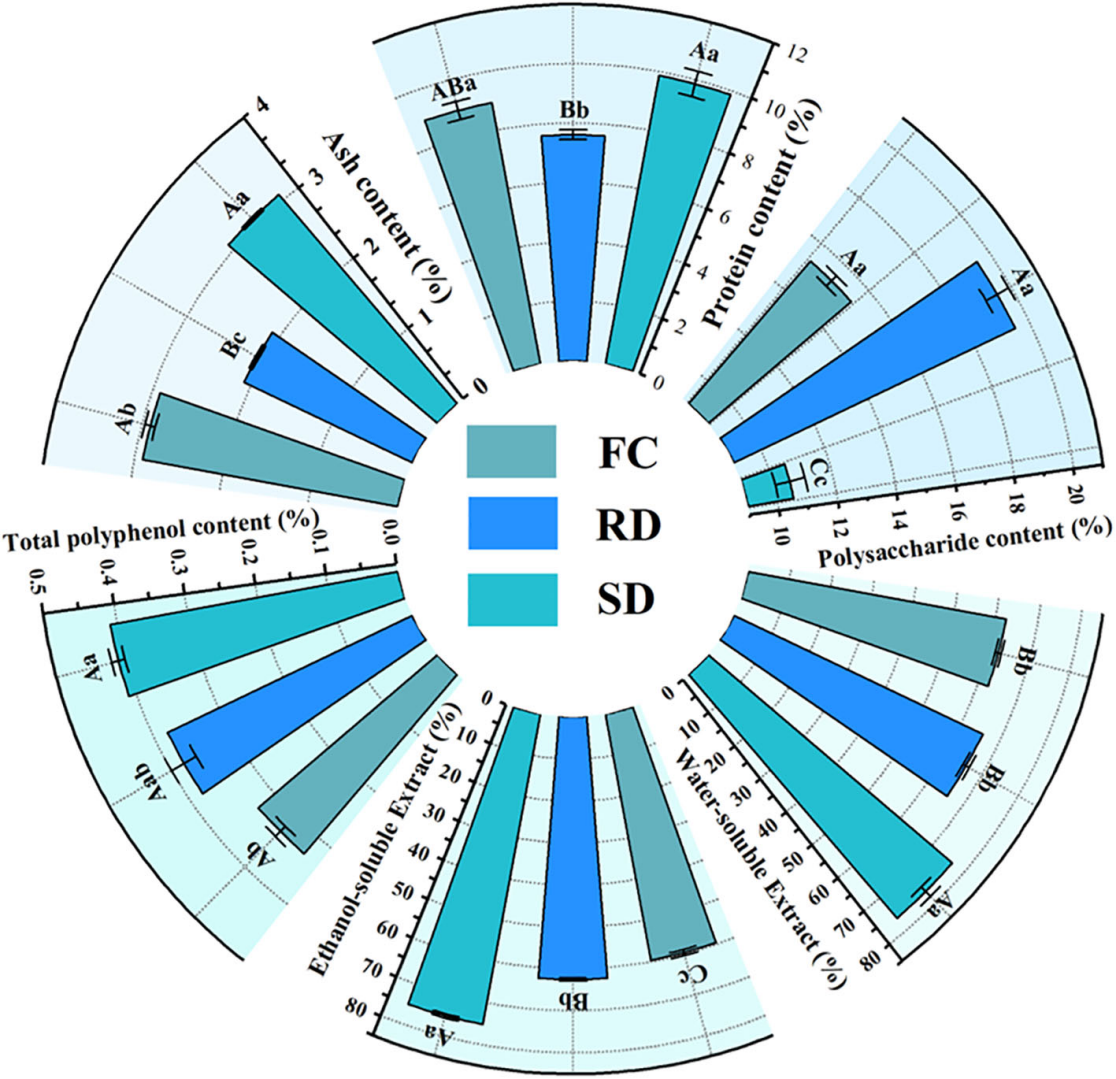
Sector bar chart of Polysaccharide (POL), protein, ash, total polyphenol (TP), ethanol-soluble extract (ASE), water-soluble extract (WSE) content in *C. pilosula.* Note: The data are presented as the means±SD. FC, Fresh *C. pilosula*; RD, “rubbing-sweating” dried *C. pilosula*; SD, Shade-dried *C. pilosula.* With in each column, the different superscripted small and capital letters Indicate significant and highly significant differences at *p*<0.05 and *p*<0.01, respectively.

The contents of polysaccharides (POL), protein, ash, total polyphenols (TP), total flavonoids (TF), ethanol-soluble extract (ASE), and water-soluble extract (WSE) in the samples are presented in **Figure 2** (**Supplementary Table S1**). The results demonstrated that different drying treatments significantly reduced the contents of protein, total phenolic compounds, ethanol-soluble extracts, and water-soluble extracts in *C. pilosula*, while significantly increasing the polysaccharide content.

Compared with the FC samples, the protein, ash, TP, ASE, and WSE contents were significantly higher in FC (9.86%, 3.08%, 0.41%, 75.16%, and 76.40%, respectively) than in RD (7.61%, 2.09%, 0.38%, 62.51%, and 65.68%, respectively) and SD (8.94%, 2.91%, 0.33%, 59.59%, and 62.48%, respectively) (*p*<0.05).

Polysaccharides, which are considered one of the primary bioactive constituents of *C. pilosula*, were notably increased by both drying treatments. The POL content in RD (19.48%) and SD (15.30%) was significantly higher than in FC (10.56%) (*p*<0.05). These results indicate that both RD and SD can enhance the accumulation of polysaccharides in *C. pilosula*, with the RD method being particularly effective.

### 3.2 Effect of dry method on SOD, POD, CAT, and APX activity

**Figure 3:**
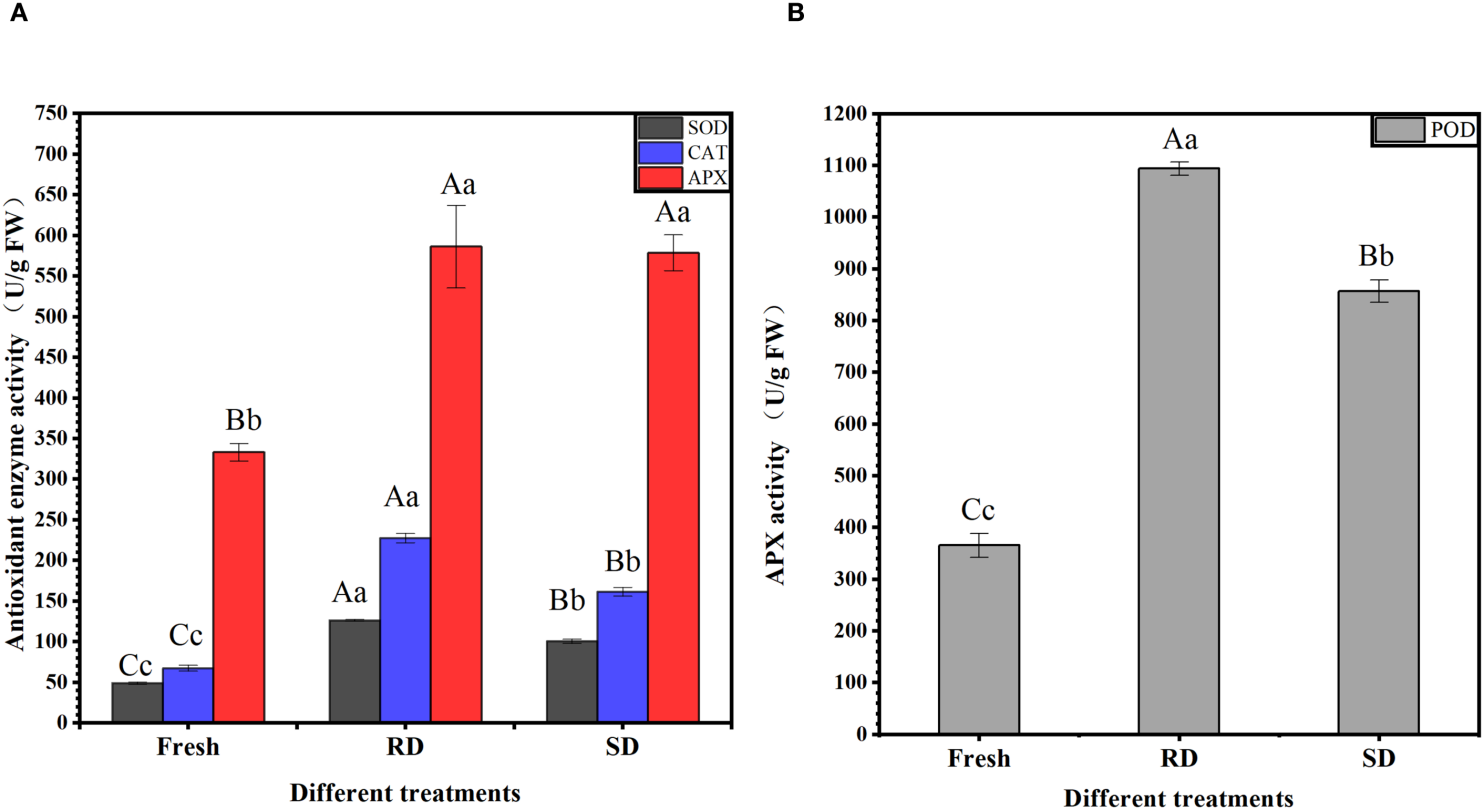
Activity of antioxidant-related enzymes. **(A)** SOD, CAT, and APX activity; **(B)** POD activity. The data are presented as the means±SD. With in each column, the different superscripted small and capital letters Indicate significant and highly significant differences at *p*<0.05 and *p*<0.01, respectively.

In “Rubbing-sweating” dried *C. pilosula* tissues, the activities of SOD and CAT reached 126.05 U/g FW and 227.42 U/g/min, respectively, which were significantly higher than those in fresh samples (49.09 U/g FW and 67.35 U/g/min) and shade-dried samples (100.38 U/g FW and 161.44 U/g/min) (*p<*0.01). The APX activities in “Rubbing-sweating” dried and shade-dried samples were 586.12 U/g FW and 578.46 U/g FW, respectively, both of which were significantly higher than that in fresh samples (333.12 U/g FW) (*p*<0.01). In addition, POD activity in “Rubbing-sweating” dried tissues reached 1093.83 U/g/min, which was significantly higher than that in fresh (365.07 U/g/min) and shade-dried (856.53 U/g/min) samples (p<0.01). SOD, APX, CAT, and POD are critical antioxidant enzymes involved in reactive oxygen species (ROS) scavenging. The significant enhancement in their activities following the RD treatment suggests that this traditional method induces more intense external stress, likely triggering increased ROS accumulation and, consequently, a stronger antioxidative response in *C. pilosula* (**Figure 3**).

### 3.3 Sequencing and sequence assembly

During the drying process, plant tissues undergo gradual senescence, accompanied by the degradation of proteins and RNA. Therefore, analyzing *C. pilosula* tissues at the early stages of drying is essential for elucidating the mechanisms underlying the accumulation of active compounds. After 6 days of drying, both RD and SD samples exhibited significant increases in active ingredient content and antioxidant enzyme activities. Consequently, transcriptomic analysis was performed on *C. pilosula* samples subjected to 6 days of RD and SD treatment to further investigate the molecular mechanisms involved in the accumulation of active compounds during the drying process.

In this study, 44,687,908, 49,800,810, and 47,962,490 clean reads were obtained from the FC, RD, and SD samples, respectively. After removing low-quality sequences, adapters, and ambiguous reads, a total of 399.11 million high-quality clean reads were acquired. These reads were assembled into 194,009 transcripts with an average length of 1,440.03 bp and an N50 length of 2,636 bp. Subsequently, the transcripts were further assembled into 102,614 unigenes, with an average length of 1,033.70 bp and an N50 length of 1,904 bp. For all samples, the mapping rate of clean reads to the reference database ranged from 85.58% to 87.56% (**Table 1**).

**Table 1:** Summary of transcriptome sequencing data and transcriptome assembly.

Sample	Raw read	Clean read	Mapped reads	Mapped ratio (%)	Error (%)	Q20 (%)	Q30 (%)
FC1	43651440	43292084	18897607	87.56	0.01	98.86	96.35
FC2	43624858	43262026	20421843	87.23	0.01	98.84	96.29
FC3	47892620	47509614	19204790	87.56	0.01	98.83	96.24
RD1	45431518	45053218	18876840	87.21	0.01	98.85	96.34
RD2	43277238	42904240	18801078	86.92	0.01	98.85	96.32
RD3	43599356	43231112	20551352	86.51	0.01	98.86	96.37
SD1	43539938	43167336	19326767	85.80	0.01	98.84	96.31
SD2	47243318	46825102	18412532	85.83	0.01	98.79	96.15
SD3	44250164	43867122	18499041	85.58	0.01	98.84	96.31
Total	402510450	399111854					
Type				Unigene		Transcript	
Total number				102614		194009	
Total base				106071857		279379336	
Largest length (bp)				18547		18547	
Smallest length (bp)				201		201	
Average length (bp)				1033.7		1440.03	
N50 length (bp)				1904		2636	
E90N50 length (bp)				3487		2975	
Fragment mapped percent(%)				61.65		87.689	
GC percent (%)				38.84		39.53	
TransRate score				0.3118		0.44594	

Raw reads: total number of sequencing reads before quality control; Clean reads: total number of sequencing reads after quality control; Mapped reads: number of clean reads successfully aligned to the assembled transcripts; Mapped ratio: percentage of paired clean reads mapped to the assembled transcripts; Error (%): average base error rate of quality-controlled data; Q20 (%): percentage of bases with a sequencing quality score higher than 99%; Q30 (%): percentage of bases with a sequencing quality score higher than 99.9%.

The contigs from the nine transcriptome sequencing datasets were integrated and assembled into a total of 102,614 unigenes. Functional annotation of these unigenes was performed using BLAST against seven public databases: GO, KEGG, eggNOG, NR, Swiss-Prot, and Pfam. A total of 30,521, 13,041, 31,191, 38,642, 21,750, and 19,871 unigenes were successfully aligned to these databases, respectively. In total, 39,037 unigenes (38.04% of all unigenes) were annotated in at least one of the functional databases (**Table 2**). This high annotation rate highlights the overall quality of the sequencing data and the effectiveness of the assembly and annotation process (Wang and Sun, 2009).

**Table 2:** Numbers of unigenes/transcript annotated using different databases.

Database	Unigene		Transcript	
	Number	Percent (%)	Number	Percent (%)
GO	30521	29.74%	88372	45.55%
KEGG	13041	12.71%	45639	23.52%
eggNOG	31191	30.40%	94486	48.70%
NR	38642	37.66%	107869	55.60%
Swiss-Prot	21750	21.20%	73421	37.84%
Pfam	19871	19.36%	66588	34.32%
Total number of annotated Unigenes/Transcript	39037	38.04%	108540	55.95%
Total number of Unigenes/Transcript	102614	100.00%	194009	100.00%

### 3.4 Identification of DEGs and cluster analysis

**Figure 4:**
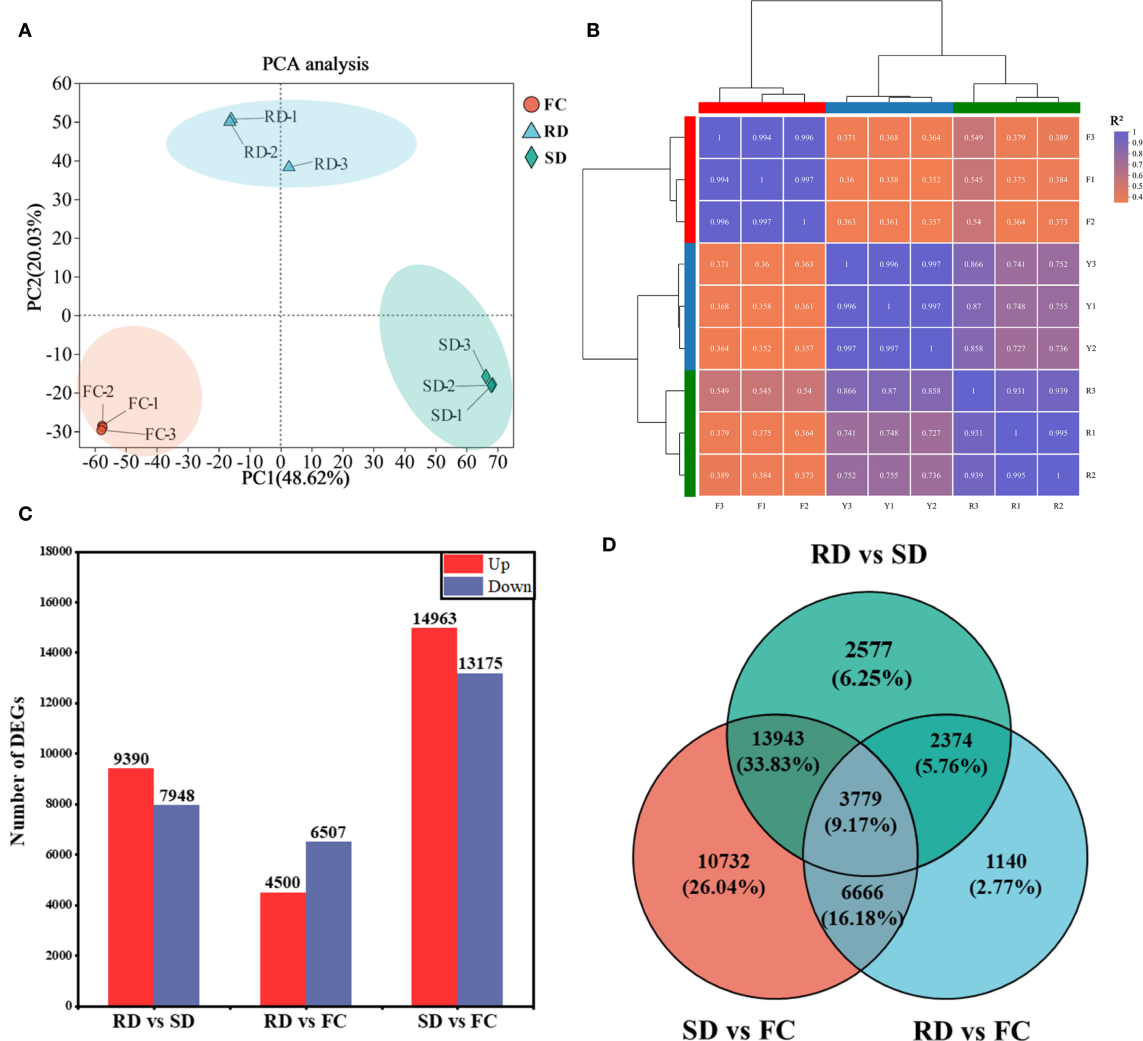
Identification of DEGs in *C*. *pilosula* samples. **(A)** Principal component analysis. **(B)** Heatmap clustering analysis and correlation coefficient of transcriptome datasets in *C*. *pilosula* samples. **(C)** Histogram of DEGs identified in *C*. *pilosula* samples. **(D)** Venn diagram of DEGs identified in *C. pilosula* samples.

Principal component analysis (PCA) revealed that the transcriptomes of FC, RD, and SD samples were clearly distinct from one another (**Figure 4A**). The correlation coefficients (R²) among the transcriptomes ranged from 0.352 to 1.000 (**Figure 4B**), indicating relatively consistent expression patterns among the nine samples and indirectly confirming the reliability of the sequencing and sampling procedures.

To identify DEGs associated with the different drying treatments, three pairwise comparisons were performed: RD vs. SD, RD vs. FC, and SD vs. FC. DEGs were defined as those with an adjusted p-value ≤ 0.05 and |log_2_ fold change| ≥ 1. A total of 17,338 DEGs (9,390 upregulated and 7,948 downregulated) were identified in RD vs. SD; 11,007 DEGs (4,500 upregulated and 6,507 downregulated) in RD vs. FC; and 28,138 DEGs (14,963 upregulated and 13,175 downregulated) in SD vs. FC (**Figure 4C**).

Among the 35,211 unique DEGs identified across all comparisons, 3,779 DEGs were shared among all three comparisons, 16,983 DEGs were shared between two comparisons, and 14,449 DEGs were specific to only one comparison (**Figure 4D**).

### 3.5 Functional analysis of DEGs with different dry process

**Figure 5:**
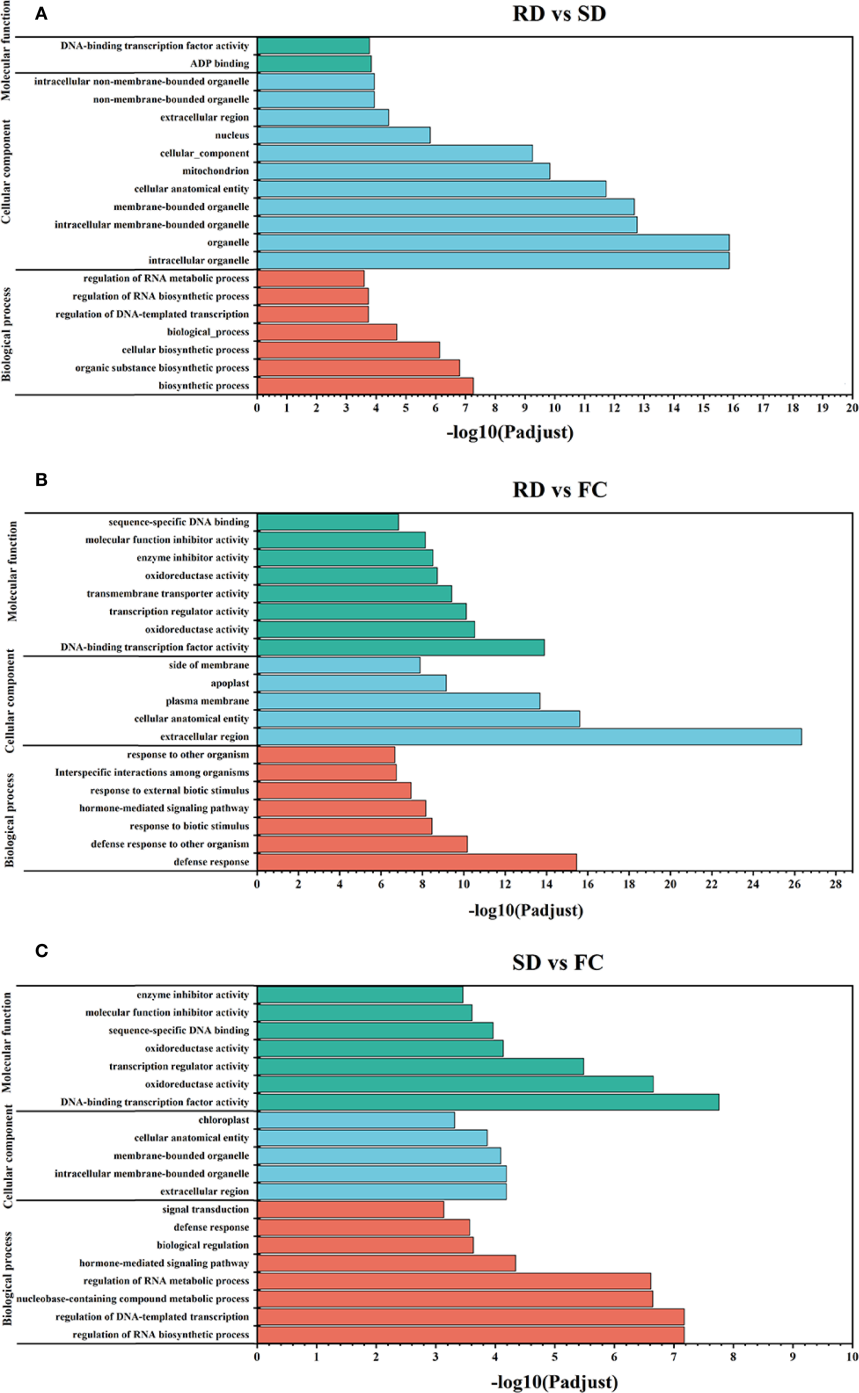
GO categories of the identified DEGs derived from *C*. *pilosula* samples. **(A)** RD vs SD; **(B)** RD vs FC; **(C)** SD vs FC.

To further elucidate the primary functions of the identified DEGs, Gene Ontology (GO) term annotation and Kyoto Encyclopedia of Genes and Genomes (KEGG) pathway enrichment analyses were conducted. According to the GO enrichment analysis results, DEGs were categorized into three main GO domains: biological processes (BP), cellular components (CC), and molecular functions (MF) (**Figure 5**). In the RD vs. SD comparison, DEGs were significantly enriched in 7 biological processes, 11 cellular components, and 2 molecular functions (**Figure 5A**). For the RD vs. FC comparison, DEGs were primarily enriched in 7 biological processes, 5 cellular components, and 8 molecular functions (**Figure 5B**). In the SD vs. FC comparison, DEGs were enriched in 8 biological processes, 5 cellular components, and 7 molecular functions (**Figure 5C**). GO enrichment analysis indicated that processes such as the regulation of RNA biosynthetic process, hormone-mediated signaling pathway, and defense response; components including the extracellular region and intracellular membrane-bounded organelles; and functions such as DNA-binding transcription factor activity, oxidoreductase activity, transcription regulator activity, and molecular function inhibitor activity were significantly associated with the DEGs. These findings suggest that these biological pathways and molecular functions may play crucial roles in metabolite transformation and the underlying molecular mechanisms of *C. pilosula* during different postharvest drying treatments.

**Figure 6:**
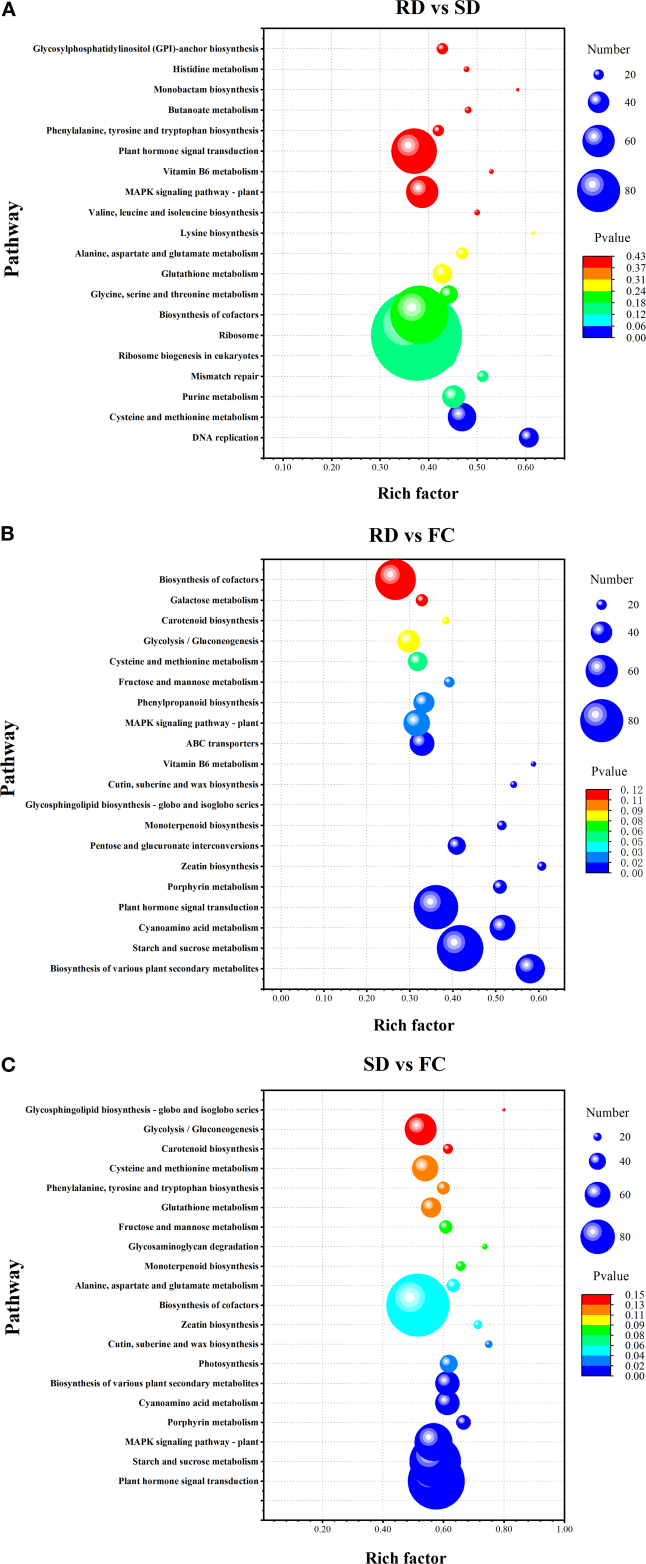
Bubble plot of the KEGG pathway enrichment statistics of DEGs. **(A)** RD vs SD; **(B)** RD vs FC; **(C)** SD vs FC.

The top 20 KEGG pathways identified from the three pairwise comparisons are illustrated in **Figure 6**. In the RD vs. SD comparison, a total of 3,554 DEGs were mapped to 134 KEGG pathways, with two pathways—DNA replication and cysteine and methionine metabolism—showing significant enrichment (**Figure 6A**). In the RD vs. FC comparison, 2,418 DEGs were enriched across 137 pathways, among which sixteen pathways exhibited significant enrichment (**Figure 6B**). These included biosynthesis of various plant secondary metabolites, starch and sucrose metabolism, plant hormone signal transduction, pentose and glucuronate interconversions, monoterpenoid biosynthesis, MAPK signaling pathway, phenylpropanoid biosynthesis, fructose and mannose metabolism, cysteine and methionine metabolism, among others.

For the SD vs. FC comparison, 5,010 DEGs were mapped to 139 pathways, with ten pathways significantly enriched (**Figure 6C**). Key enriched pathways included plant hormone signal transduction, starch and sucrose metabolism, MAPK signaling pathway, and biosynthesis of various plant secondary metabolites. These findings suggest that metabolic and signaling pathways related to hormone regulation, carbohydrate metabolism, and secondary metabolite biosynthesis play pivotal roles in mediating the effects of traditional postharvest drying methods on the quality and bioactivity of *C. pilosula*. These pathways likely contribute to the enhanced accumulation of active compounds observed following rubbing-sweating and shade drying treatments.

### 3.6 qRT-PCR-based verification

**Figure 7:**
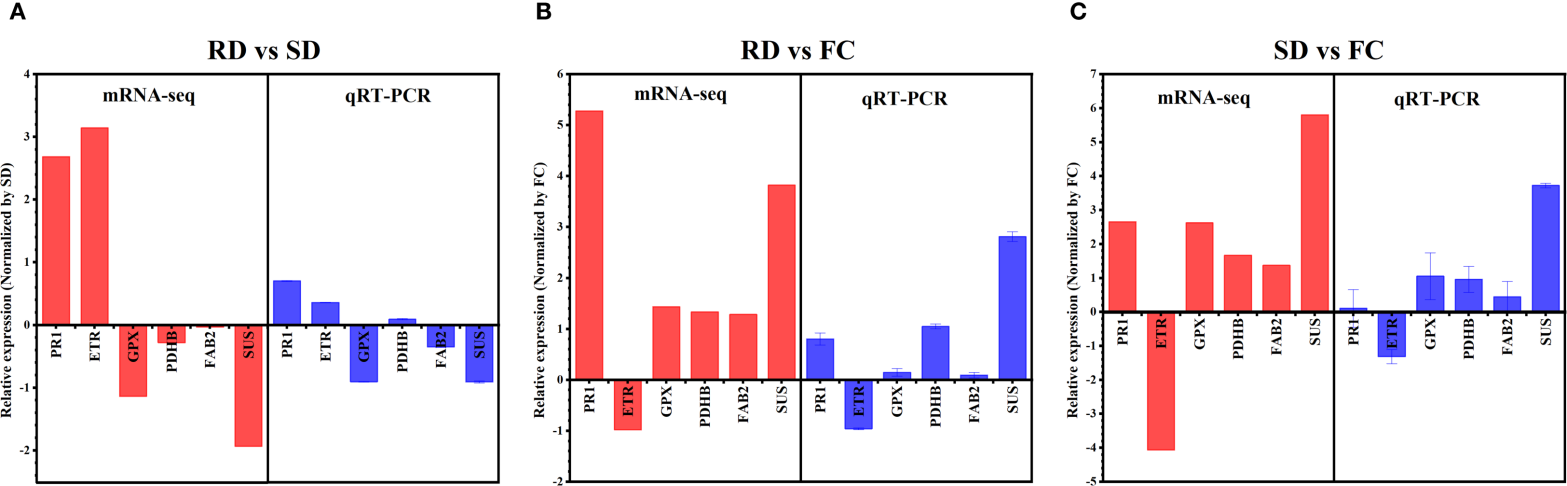
Validation of the expression patterns of DEGs selected from the RNA-Seq analysis by qRT-PCR. **(A)** AD versus SD; **(B)** RD versus FC; **(C)** SD versus FC. RP-1, pathogenesis-related protein 1; ETR, Ethylene receptor; gpx, glutathione peroxidase; PDHB, putative pyruvate dehydrogenase; FAB2, acyl-[acyl-carrier protein] desaturase; SUS, sucrose synthase.

To validate the RNA-sequencing (RNA-Seq) results, six DEGs RP-1 (pathogenesis-related protein 1), ETR (Ethylene receptor), GPX (glutathione peroxidase), PDHB (putative pyruvate dehydrogenase), FAB2 (acyl-[acyl-carrier protein] desaturase), SUS (sucrose synthase) related to *C. pilosula* substance stress response, signal transduction and metabolism were selected for qRT-PCR analysis. The results showed that The mRNA-Seq and RT-PCR data were very closely correlated, and there was high consistency in the up- and down-regulated expression of DEGs. This result supports the reliability of the RNA-Seq analysis, indicating that transcription data were accurate and effective and can be used for the gene expression profile analysis in different drying processes of *C. pilosula* (**Figure 7**).

## 4 Discussion

### 4.1 Differences in chemical composition and antioxidant enzyme activity of *C. pilosula* under different drying treatments

Significant differences in the chemical and nutritional compositions of *C. pilosula* were observed under different drying methods (**Figures 1** and **2**). Compared with FC, both SD and RD significantly increased the contents of bioactive constituents (*p*<0.01), with the increase being more pronounced in RD. In particular, the polysaccharide and lobetyolin contents in RD reached 19.50% and 141.18 μg/g, respectively, which were significantly higher than those in FC (10.56% and 122.44 μg/g) and SD (15.30% and 128.67 μg/g) (*p*<0.01). These results indicate that RD facilitates the accumulation of major active constituents, which may be associated with stress-induced activation of secondary metabolism. This finding is consistent with previous reports that postharvest processing methods markedly improve the quality of other medicinal materials, such as rhubarb and Radix Gentianae (Liang et al., 2021, 2022; Sun et al., 2023), further supporting the notion that abiotic stress can enhance the biosynthesis and accumulation of key active compounds in medicinal plants.

Analysis of antioxidant enzyme activities showed that after 6 days of RD and SD treatment, the activities of SOD, CAT, APX, and POD were all significantly elevated (*p*<0.01), indicating that both drying methods triggered oxidative stress responses in *C. pilosula* tissues. Notably, the activities of SOD, CAT, and POD in RD were significantly higher than those in SD (*p*<0.01), suggesting that RD induced a stronger stress stimulus, leading to more pronounced oxidative stress and activation of antioxidant defense systems. This enhanced stress response may be closely related to the more substantial accumulation of secondary metabolites observed under RD.

In summary, rubbing–sweating drying significantly increased the contents of active constituents such as polysaccharides and lobetyolin and markedly enhanced antioxidant enzyme activities. These findings indicate that this drying method improves the medicinal quality of *C. pilosula* by inducing stress responses and activating relevant metabolic pathways. To systematically elucidate the molecular mechanisms by which different drying methods affect the quality of medicinal materials, transcriptomic analysis was performed on day 6, when changes in active constituents and antioxidant enzyme activities were most pronounced.

### 4.2 Differential gene expression related to stress response

Reactive oxygen species (ROS) are oxygen-containing molecules with higher chemical reactivity than molecular oxygen and act as double-edged regulators in plants (Castro et al., 2021). Excessive ROS accumulation damages proteins, lipids, and nucleic acids, leading to cell death, whereas controlled ROS production functions as an essential signal to coordinate responses to biotic and abiotic stress (Choudhury et al., 2017). The major ROS in plant cells—singlet oxygen, superoxide anions, hydrogen peroxide, and hydroxyl radicals—are primarily generated in chloroplasts, mitochondria, and peroxisomes (Mittler, 2017; Mittler et al., 2022). Antioxidant defenses, including enzymatic (SOD, CAT, POD, APX) and non-enzymatic pathways such as the ascorbate-glutathione (AsA-GSH) cycle, maintain redox homeostasis (Hasanuzzaman et al., 2020; Tai et al., 2022; Li, 2023).

**Figure 8:**
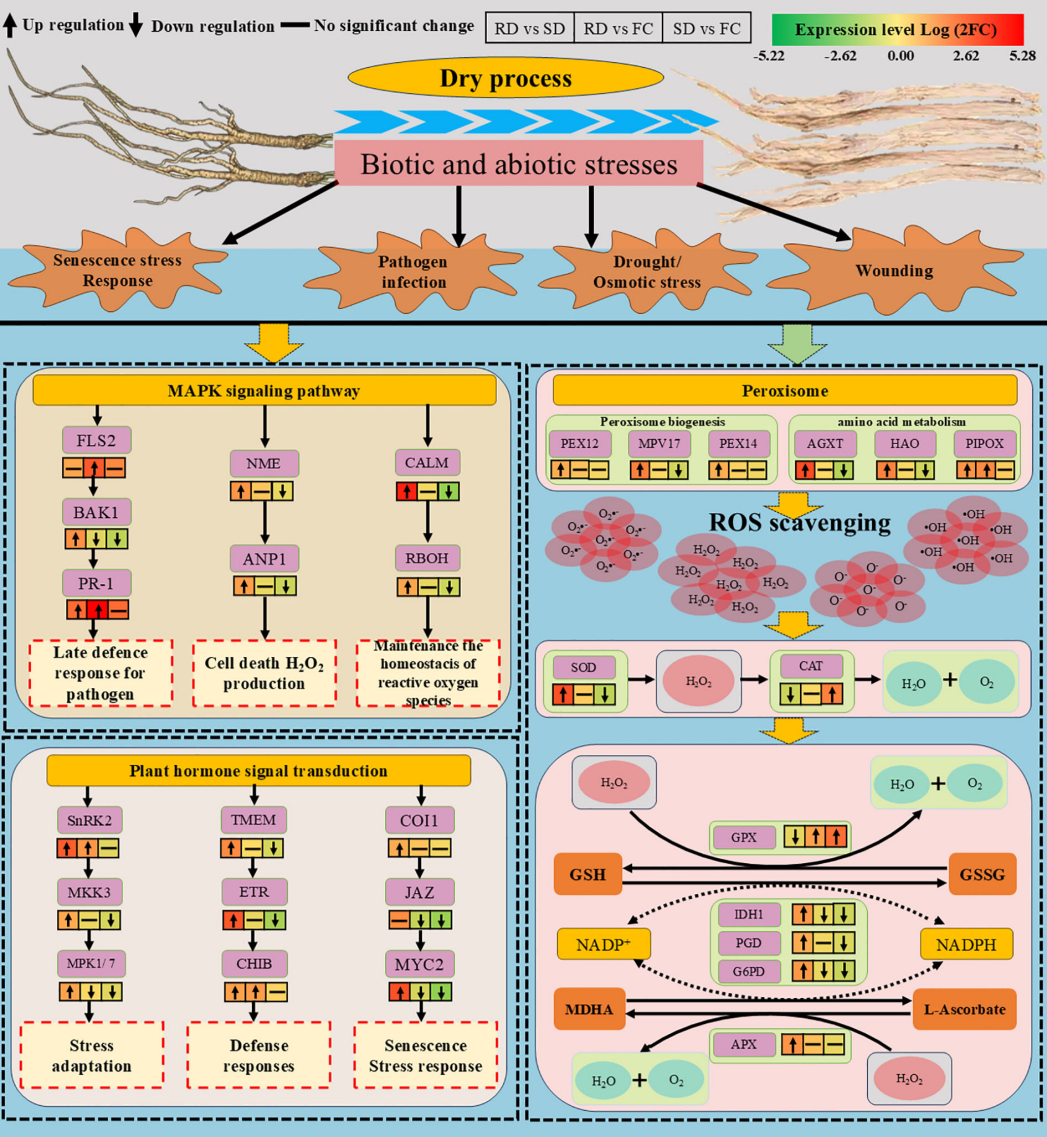
Overview of DEGs related to stress response and signal transduction during the drying process of *C. pilosula.* (FLS2, LRR receptor-like serine/threonine-protein kinase FLS2; BAK1, brassinosteroid insensitive 1-associated receptor kinase 1; PR-1, pathogenesis-related protein 1; NME, nucleoside-diphosphate kinase;ANP1, mitogen-activated protein kinase kinase kinase ANP1; CALM, calmodulin; RBOH, respiratory burst oxidase; SnRK2, serine/threonine-protein kinase SAPK7-like; MKK3, mitogen-activated protein kinase kinase 3; MPK1/7, mitogen-activated protein kinase 1/7; TMEM, transmembrane protein; ETR, ethylene receptor; CHIB, endochitinase A; COI1, coronatine insensitive 1; JAZ, protein TIFY 6B isoform X1; MYC2, transcription factor MYC2; PEX12, peroxin-12; MPV17, protein Mpv17; PEX14, peroxin-14; AGXT, serine–glyoxylate aminotransferase; HAO, FMN-dependent dehydrogenase; PIPOX, sarcosine oxidase/L-pipecolate oxidase; SOD, superoxide dismutase; CAT, catalase; GPX, glutathione peroxidase; IDH1, NADP-isocitrate dehydrogenase); PGD, 6-phosphogluconate dehydrogenase; G6PD, glucose-6-phosphate 1-dehydrogenase; APX, ascorbate peroxidase).

Peroxisomes play a particularly important role in regulating oxidative metabolism. Peroxins, which are essential for peroxisome biogenesis and function, maintain peroxisomal integrity and mediate stress responses (Traver et al., 2022; Collin and Daszkowska-Golec, 2025). In this study, the transcriptional levels of *PEX12* and *PEX14* were significantly upregulated in RD vs. SD, suggesting enhanced peroxisomal activity under rubbing-sweating treatment. Meanwhile, *MPV17*, a gene encoding a peroxisomal membrane protein involved in ROS generation (Chen et al., 2024), was upregulated in RD vs. SD but downregulated in SD vs. FC, indicating that RD imposes stronger oxidative stress than SD. Furthermore, genes encoding peroxisomal enzymes such as glyoxylate aminotransferase (*AGXT*) and hydroxyacid oxidase (*HAO*), both of which contribute to adaptation under drought and salt stress (Hu et al., 2012; Zeier, 2013; Barqawi and Abulfaraj, 2023), were significantly upregulated in RD vs. SD, but downregulated in SD vs. FC. In addition, *PIPOX*, a key enzyme regulating pipecolic acid metabolism and plant immunity (Zeier, 2013; Chen et al., 2018), was markedly upregulated in both RD vs. SD and RD vs. FC, while no changes were observed in SD vs. FC. Together, the coordinated upregulation of *PEX12*, *PEX14*, *MPV17*, *AGXT*, *HAO*, and *PIPOX* in RD vs. SD supports the conclusion that rubbing-sweating imposes greater abiotic stress on *C. pilosula* tissues, triggering peroxisome-related pathways and promoting ROS generation that likely activates secondary metabolism (**Figure 8**).

Plants employ a range of antioxidant systems to mitigate ROS toxicity. SOD catalyzes the dismutation of superoxide radicals to H_2_O_2_, which is further decomposed by CAT and POD (Hasanuzzaman et al., 2020). In this study, genes encoding SOD were upregulated in RD vs. SD but downregulated in SD vs. FC, indicating a stronger oxidative response under RD. POD and CAT showed similar trends, supporting the notion of enhanced ROS detoxification capacity under RD treatment. The AsA-GSH cycle represents a critical pathway for maintaining redox balance in chloroplasts, mitochondria, and cytosol (Tai et al., 2022). Ascorbate peroxidase (APX) plays a complementary role by catalyzing the reduction of H_2_O_2_ using ascorbate (AsA) as an electron donor, producing monodehydroascorbate (MDHA) and H_2_O (Zhang et al., 2023; Caccamo et al., 2024). Within this cycle, glutathione peroxidase (GPX) reduces H_2_O_2_ using GSH, which is regenerated via NADPH-dependent reactions. In this study, GPX-related genes were significantly upregulated in both RD vs. FC and SD vs. FC, suggesting activation under both drying conditions. However, *APX* genes were particularly upregulated in RD vs. FC, reinforcing the specific contribution of the AsA-GSH cycle to ROS scavenging under rubbing-sweating treatment. Importantly, genes encoding NADPH-generating enzymes such as *IDH1*, *PGD*, and *G6PD* were significantly upregulated in RD vs. SD. These enzymes are not only critical for regenerating reduced glutathione and ascorbate (Huang et al., 2019; Hasanuzzaman et al., 2020), but also central to the TCA cycle and pentose phosphate pathway, thereby contributing to enhanced energy production and precursor supply. Their upregulation indicates that RD treatment boosts metabolic capacity to support both redox homeostasis and secondary metabolite biosynthesis (**Figure 8**).

Taken together, these results demonstrate that RD imposes stronger oxidative stress on *C. pilosula* than SD, as evidenced by elevated ROS generation and coordinated activation of peroxisome-associated metabolic pathways, antioxidant enzymes, and the AsA-GSH cycle. This enhanced stress response not only maintains cellular redox balance but also drives increased metabolic activity and the accumulation of bioactive compounds, thereby improving the medicinal quality of *C. pilosula*.

### 4.3 Differential gene expression related to signal transduction

Signal transduction is essential for plants to perceive environmental stimuli and activate appropriate physiological and molecular responses, thereby enhancing stress tolerance (Manna et al., 2023). Among the various pathways, the mitogen-activated protein kinase (MAPK) cascade plays a central role in mediating responses to both biotic and abiotic stresses. In this study, DEGs within the MAPK pathway were mainly associated with defense activation, H_2_O_2_ production, and the regulation of ROS homeostasis.

In plant immunity, the receptor FLS2 specifically recognizes pathogen-associated peptides such as flg22, with BAK1 serving as a co-receptor to initiate downstream immune signaling (Yuan et al., 2021; Wang et al., 2024). PR-1, a key defense protein, subsequently accumulates to inhibit pathogen proliferation (Zhang et al., 2022b). Here, BAK1 and PR-1 were significantly upregulated in RD vs. SD, while FLS2 and PR-1 were enhanced in RD vs. FC, suggesting that mechanical stress during rubbing-sweating (RD) may disrupt cell walls, enhance PAMP (pathogen-associated molecular patterns) perception, and activate immune signaling. NDPK2 and ANP1 further regulate the MAPK cascade. NDPK2 promotes H_2_O_2_ accumulation and programmed cell death (Liu et al., 2025), while ANP1, as a MAPKKK, responds to oxidative stress to activate downstream MAPK (Marti et al., 2021). Both were upregulated in RD vs. SD but downregulated in SD vs. FC, indicating that RD strongly induced oxidative signaling and stress-related cell death in *C. pilosula* tissues. Calcium-mediated signaling also plays a crucial role. Calmodulin (CALM) functions as a Ca²^+^ sensor, regulating RbohD activity, which transfers electrons from NADPH to oxygen to generate ROS (Dubiella et al., 2013; Li et al., 2014; Seybold et al., 2014). In this study, CALM and RbohD were significantly upregulated in RD vs. SD, implying that RD enhanced Ca²^+^ influx and ROS production. Meanwhile, MYC2-related genes were downregulated in SD vs. FC, indicating differential activation of jasmonate signaling under shade drying. Together, these results suggest that RD treatment imposed stronger abiotic stress, triggering Ca²^+^–ROS signaling and potentially regulating secondary metabolism (**Figure 8**).

Plant hormone signaling further contributes to stress responses. In the abscisic acid (ABA) pathway, SnRK2 mediates responses to drought, salinity, and other stresses (Zhao et al., 2023). In this study, SnRK2, MKK3, and MPK1 were significantly upregulated in RD vs. SD, while MKK3 and MPK1 were downregulated in SD vs. FC. As MKK3 activates downstream MAPKs such as MPK1 and MPK7 to regulate disease resistance (Zhou et al., 2019a; Li et al., 2022), these results indicate that RD imposed stronger stress, thereby enhancing ABA- and MAPK-mediated signaling. Ethylene signaling was also activated under RD. RTE1 regulates the receptor ETR1, which controls CTR1 activity to initiate downstream signaling (Chen et al., 2022b). Both RTE1 and ETR1 were significantly upregulated in RD vs. SD, suggesting enhanced ethylene signaling. Ethylene mediates responses to diverse stresses, including drought, temperature, salinity, and mechanical damage (Pérez-Llorca et al., 2023). Moreover, ChiB, a chitinase gene related to ethylene-mediated defense, was upregulated in both RD vs. SD and RD vs. FC, further supporting the view that the RD process significantly enhances the defense capacity of *C. pilosula* tissues (Vaghela et al., 2022). Jasmonate (JA) signaling also responded strongly to RD. In this pathway, mechanical stress increases JA levels, promoting COI1–JA receptor complex formation, degradation of JAZ repressors, and release of MYC2 to activate defense-related and senescence-associated genes (Zhou et al., 2019b). In our study, COI1 and MYC2 were upregulated in RD vs. SD, while MYC2 was suppressed in SD vs. FC. These findings suggest that RD activated JA signaling more effectively than SD, enhancing stress resistance but potentially accelerating senescence (**Figure 8**).

Taken together, transcriptome data demonstrate that rubbing-sweating more strongly activates signal transduction pathways than shade drying. RD treatment upregulated multiple genes in MAPK signaling and hormone pathways, including BAK1, PR-1, NDPK2, ANP1, CALM, RbohD, SnRK2, MKK3, MPK1, RTE1, ETR1, ChiB, COI1, and MYC2. This indicates that RD imposes stronger abiotic stresses—mechanical damage, hypoxia, and elevated temperature—thereby stimulating immune responses, ROS production, and hormone signaling. Such activation likely enhances stress adaptation and secondary metabolite accumulation, contributing to improved medicinal quality of *C. pilosula*. Nevertheless, the pronounced stress response may also accelerate senescence and programmed cell death, reflecting a trade-off between enhanced quality and tissue longevity under traditional rubbing-sweating processing.

### 4.4 Differential gene expression related to phenylalanine, tyrosine and tryptophan biosynthesis

*C. pilosula*, a traditional medicinal and edible herb, is rich in amino acids that contribute significantly to its nutritional and pharmacological value (Luan et al., 2021). Among them, the aromatic amino acids (AAAs)—L-tryptophan (Trp), L-phenylalanine (Phe), and L-tyrosine (Tyr)—are not only indispensable for protein synthesis but also serve as precursors for diverse natural products, influencing plant growth, defense, and stress responses (Yokoyama, 2024). In this study, multiple genes involved in AAA biosynthesis were differentially expressed under different drying treatments. Their biosynthesis occurs in two main stages: the conversion of D-erythrose 4-phosphate into chorismate via the shikimate pathway, followed by chorismate-mediated branching into distinct downstream pathways. The shikimate pathway also generates antioxidants such as flavonoids, phenolics, and lignin, which mitigate oxidative stress by scavenging ROS (Tohge et al., 2013; Zhao et al., 2018). Within this pathway, aroF, aroB, and aroDE encode key enzymes that catalyze the formation of intermediates including 3-dehydroshikimate and chorismate. Notably, 3-dehydroquinate synthase (aroB) regulates protocatechuic acid biosynthesis, a phenolic compound that reduces ROS levels and enhances stress resilience (Hao et al., 2022; Zhang et al., 2022a). In wheat, aroDE is upregulated under drought and salinity stress (Dugasa et al., 2020). Here, aroF, aroB, and aroDE were significantly upregulated in RD vs. SD, suggesting that rubbing-drying imposes stronger mechanical, hypoxic, and dehydration stresses than SD. This activation likely promotes chorismate synthesis, driving downstream AAA biosynthesis and contributing to secondary metabolite accumulation.

**Figure 9:**
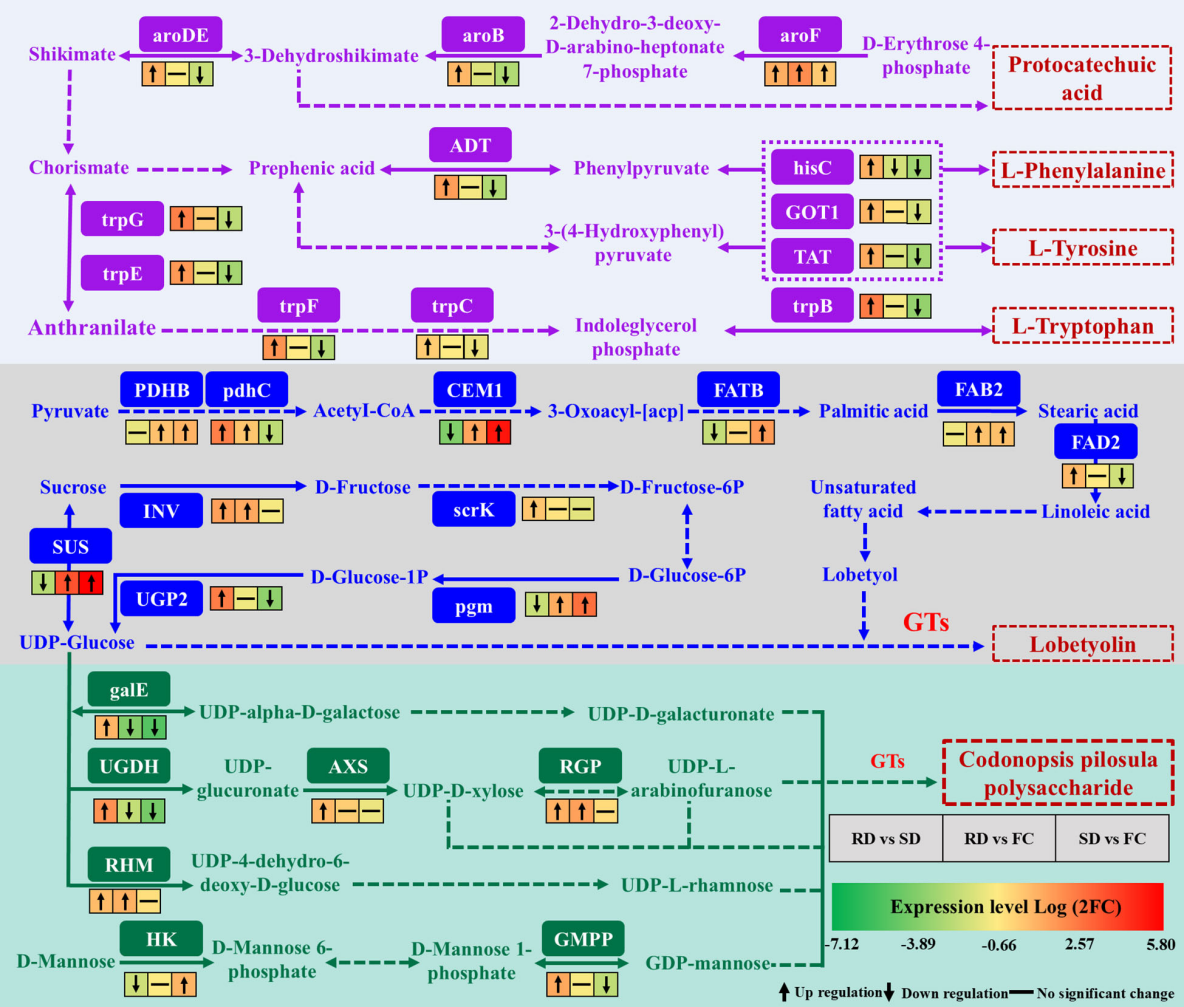
Overview of DEGs related to phenylalanine, tyrosine and tryptophan biosynthesis during the drying process of *C. pilosula.* (aroDE, 3-dehydroquinate dehydratase/shikimate dehydrogenase; aroB, 3-dehydroquinate synthase; aroF, phospho-2-dehydro-3-deoxyheptonate aldolase 2; ADT, arogenate/prephenate dehydratase; hisC, histidinol-phosphate aminotransferase; GOT1, aspartate aminotransferase; TAT, tyrosine aminotransferase-like isoform X1; trpG, anthranilate synthase beta subunit 1; trpE, anthranilate synthase alpha subunit 2; trpF, N-(5’-phosphoribosyl)anthranilate isomerase 1; trpC, anthranilate synthase/indole-3-glycerol phosphate synthase; trpB, tryptophan synthase alpha chain; PDHB, putative pyruvate dehydrogenase; pdhc, pyruvate dehydrogenase E2 component; CEM1, 3-oxoacyl-[acyl-carrier-protein] synthase II; FATB, palmitoyl-acyl carrier protein thioesterase; FAB2, acyl-[acyl-carrier protein] desaturase; FAD2, Delta(12) fatty acid desaturase DES8.11; SUS, sucrose synthase; INV, beta-fructofuranosidase; scrK, fructokinase-6; UGP2, UTP–glucose-1-phosphate uridylyltransferas; pgm, phosphoglucomutase; galE, UDP-glucose 4-epimerase; UGDH, UDPglucose 6-dehydrogenase; AXS, UDP-apiose/xylose synthase; RGP, UDP-arabinopyranose mutase; RHM, UDP-glucose 4,6-dehydratase; HK, hexokinase; GMPP, mannose-1-phosphate guanylyltransferase).

Phe and Tyr are not only structural amino acids but also precursors of diverse polyphenols with antioxidant properties (Gechev et al., 2013; Sun et al., 2023). Both are implicated in activating ROS-scavenging systems and alleviating oxidative damage (Ramzan et al., 2023). In this study, genes including ADT, hisC, GOT1, and TAT—encoding key enzymes for Phe and Tyr biosynthesis—were significantly upregulated in RD vs. SD, unchanged in RD vs. FC, and downregulated in SD vs. FC. These patterns indicate that RD more effectively enhances Phe/Tyr biosynthesis, potentially improving ROS detoxification and stress tolerance in *C. pilosula* (**Figure 9**).

For Trp biosynthesis, anthranilate synthase (trpG and trpE) catalyzes the conversion of chorismate to anthranilate, initiating the pathway. These genes are known to regulate root stress responses and development (Tu et al., 2021). Shade treatment upregulates trpG in walnut, increasing protein accumulation (Liang et al., 2023). Other genes such as trpF, trpC, and trpB also participate in Trp synthesis (Maeda and Dudareva, 2012). In our study, trpG, trpE, trpF, trpC, and trpB were significantly upregulated in RD vs. SD but downregulated in RD vs. FC, suggesting enhanced transcriptional activation of the Trp pathway under rubbing stress. However, despite this upregulation, Trp content was higher in SD than RD. This discrepancy may reflect accelerated Trp turnover under stress: as a precursor for indole-derived metabolites such as auxin (IAA), serotonin, and alkaloids, Trp is rapidly consumed during stress responses (Ljung, 2013; Ren et al., 2025). Thus, RD may promote higher flux through the Trp pathway but reduce net accumulation due to enhanced conversion into secondary metabolites (**Figure 9**).

Chorismate and its derivatives Phe, Tyr, and Trp are central intermediates in numerous physiological processes, serving as precursors of lignin, flavonoids, and auxins while sustaining metabolic homeostasis and stress adaptation (Shende et al., 2024; El-Azaz and Maeda, 2025). Our findings show that RD strongly upregulated genes across this pathway, indicating that mechanical and hypoxic stresses activate AAA biosynthesis and promote secondary metabolite production. This enhanced metabolic activity may increase the nutritional and medicinal value of *C. pilosula*. Overall, the results highlight the dual role of RD-induced stress: while it activates key biosynthetic pathways, leading to enhanced accumulation of secondary metabolites and potential improvement in medicinal quality, it also accelerates metabolic turnover of certain compounds such as Trp. Therefore, the RD process should be carefully optimized—by adjusting kneading cycles and sweating duration—to balance stress-induced quality enhancement with the risk of excessive nutrient loss.

### 4.5 Differential gene expression related to lobetyolin biosynthesis

Lobetyolin, a characteristic polyacetylene compound in *C. pilosula*, exhibits diverse pharmacological activities, including antioxidant, anti-inflammatory, and immunomodulatory effects, and is therefore regarded as an important quality marker (He et al., 2020; Xie et al., 2023). However, its biosynthetic pathway remains poorly characterized. Previous studies have suggested that lobetyolin may derive from pyranose-form glucose metabolism (Bailly, 2021) or originate from oleic acid through fatty acid metabolism, with intermediates such as citric acid and linoleic acid serving as potential precursors (Xu et al., 2024). Comparative analyses among *C. pilosula* varieties further indicate that lobetyolin likely consists of a fatty acid chain conjugated with a glycosyl moiety, reinforcing the role of fatty acid metabolism in its biosynthesis (Aghkand et al., 2019; Ma et al., 2024). In the putative pathway, pyruvate is converted into acetyl-CoA by PDHB and pdhC, which then enters fatty acid metabolism. In this study, the genes encoding PDHB and pdhC were significantly upregulated in the RD vs. FC comparison, suggesting enhanced acetyl-CoA supply under rubbing-drying. Acetyl-CoA is subsequently elongated and modified through enzymes such as CEM1 and FATB, leading to palmitic acid formation, which can be further desaturated by FAB2 to oleic acid. Oleic acid is converted by FAD2 to linoleic acid, a key intermediate for polyacetylene biosynthesis (Xu et al., 2024). Here, CEM1 was significantly upregulated in RD vs. FC, while both CEM1 and FATB showed strong induction in SD vs. FC, indicating that fatty acid metabolism is activated by both drying methods, though via slightly different patterns. FAB2 expression was significantly upregulated in both RD vs. FC and SD vs. FC, while FAD2 was higher in RD vs. SD, suggesting that RD more strongly promotes the conversion of oleic acid to linoleic acid. Taken together, these results suggest that lobetyolin biosynthesis in *C. pilosula* is closely linked to fatty acid metabolism, particularly the conversion of acetyl-CoA through sequential desaturation steps leading to linoleic acid. Both SD and RD enhanced the expression of key genes in this pathway, but RD imposed stronger stress stimuli, thereby exerting a more pronounced promotive effect on lobetyolin accumulation (**Figure 9**).

In addition to unsaturated fatty acid chains, lobetyolin biosynthesis requires glycosylation mediated by glycosidic compounds (Xu et al., 2024). Within the starch and sucrose metabolism pathway, sucrose is converted into UDP-glucose through the actions of INV, scrK, pgm, UGP2, and SUS, providing the essential glycosyl donor. UDP-glucose is subsequently conjugated to lobetyol by glycosyltransferases (GTs), yielding lobetyolin. In this study, pgm and SUS were significantly upregulated in both RD vs. FC and SD vs. FC, while INV, scrK, and UGP2 showed higher expression in RD vs. SD. These results indicate that RD more strongly stimulates UDP-glucose biosynthesis and related glycosylation processes than SD, thereby facilitating lobetyolin formation (**Figure 9**).

Overall, lobetyolin biosynthesis in *C. pilosula* is closely associated with genes from the TCA cycle, fatty acid metabolism, and starch and sucrose metabolism. Transcriptomic analysis demonstrated that drying methods differentially regulate these pathways, with rubbing-drying exerting a stronger promotive effect. These transcriptomic results were consistent with quantitative content determination (**Figure 1**), confirming that rubbing-drying enhances lobetyolin accumulation in *C. pilosula*.

### 4.6 Differential gene expression related to *C. pilosula* polysaccharide biosynthesis

The biosynthesis of polysaccharides in plants mainly involves starch/sucrose and amino sugar/nucleotide sugar metabolism. Sucrose is first converted into UDP-glucose by sucrose synthase (SUS), which provides precursors for multiple nucleotide sugars. For example, GalE forms UDP-D-galacturonate, UGDH produces UDP-glucuronic acid, AXS generates UDP-D-xylose, RGP forms UDP-L-arabinofuranose, and RHM synthesizes UDP-L-rhamnose. These activated sugars are incorporated into polysaccharide chains by GTs, contributing to cell wall architecture, intracellular signaling, and stress responses (Wang et al., 2017; Niu et al., 2020; Zhang et al., 2020). Alternatively, sucrose can also be metabolized by SUS and HK into D-mannose-6-phosphate, then converted into GDP-mannose by GMPP, which also functions as a sugar donor in polysaccharide biosynthesis.

In this study, genes encoding RGP and RHM were significantly upregulated in RD vs. FC, while AXS, HK, and GMPP showed an upward trend. Furthermore, galE, UGDH, AXS, RGP, RHM, and GMPP were significantly upregulated in RD vs. SD, suggesting that RD promotes polysaccharide biosynthesis more effectively than SD or FC (**Figure 9**). Previous studies support this view: UGDH is induced by drought in barley (Vitámvás et al., 2015), AXS enhances oxidative stress resistance in rice (Ni et al., 2022), RGP contributes to stress defense (Saqib et al., 2019), and GMPP improves salt tolerance in rice (Chen et al., 2022a). These results indicate that RD imposes stronger abiotic stress, including mechanical injury, pathogen exposure, hypoxia, and elevated temperature, thereby stimulating polysaccharide accumulation in *C. pilosula*.

## 5 Conclusion

In this study, the differences in key active compounds in *C. pilosula* under shade drying and rubbing–sweating drying were systematically analyzed. For the first time, the molecular mechanisms underlying the accumulation of these compounds during postharvest processing were explored using transcriptomic approaches. The results demonstrated that both drying methods enhanced the quality of *C. pilosula* to varying degrees, with rubbing–sweating being more conducive to the accumulation of major active constituents such as lobetyolin and polysaccharides.

Transcriptomic analysis further revealed that rubbing–sweating more strongly activated the expression of genes involved in MAPK signaling and hormone transduction, thereby inducing stress responses, defense mechanisms, and programmed cell death. In addition, peroxisome-related pathways and the antioxidant enzyme system were markedly upregulated, promoting redox homeostasis and the biosynthesis of secondary metabolites. Compared with SD, RD also enhanced the expression of genes related to phenylalanine, tyrosine, tryptophan, lobetyolin, and polysaccharide biosynthesis, suggesting that the enhanced quality results from stress-induced activation of multiple metabolic pathways.

Although this study, for the first time, provides transcriptomic evidence linking the rubbing and sweating treatment with the improvement of *C. pilosula* quality, further in-depth investigation is still required. Future research will integrate multi-omics data to conduct joint analyses of DEGs and metabolites during the drying process of *C. pilosula*, as well as perform functional validation of key genes involved in critical pathways. On this basis, systematic optimization of key processing parameters in producing areas (such as rubbing intensity, rubbing frequency, sweating duration, and environmental conditions) will be undertaken to establish technical standards for *C. pilosula* processing, thereby standardizing the processing workflow. This will provide both scientific evidence and practical guidance to promote the modernization and mechanization of *C. pilosula* processing.

## Data Availability

The data presented in the study are deposited in the CNCB-NGDC Genome Sequence Archive (GSA), BioProject accession number PRJCA039682 (https://ngdc.cncb.ac.cn/gsa/browse/CRA025395).
