# Supplementary material for: Effects of post-harvest ‘ rubbing-sweating ‘ drying treatment on the accumulation of bioactive compounds in Codonopsis pilosula: a transcriptomic analysis
Source: Front Plant Sci. 2025 Sep 30;16:1650787. doi: 10.3389/fpls.2025.1650787 (PMC12518347; doi:10.3389/fpls.2025.1650787)
Supplement: Supplementary file 1 [file Table1.docx]

Supplementary Material

Effects of Post-Harvest ' Rubbing-sweating ' Drying Treatment on the Accumulation of Bioactive Compounds in *Codonopsis pilosula*: A Transcriptomic Analysis

Wei Liang ^1^*, Gang Bai ^1^, Jiachen Sun ^2^, Wenzhen Tao^3^,Qian Li ^1^, Pengbin Dong ^1^, HongyanWang ^1^, Jiali Cheng ^1^, Fengxia Guo ^1^*, Yuan Chen ^1^*

^1^ State Key Laboratory of Arid land Crop Science, College of Agronomy, College of Life Science and Technology, Gansu Agricultural University, Lanzhou 730070, China

^2^ School of Biotechnology and Food Science, Tianjin University of Commerce, Tianjin 300134, China

^3^ Jingyuan Road Community Health Center Chengguan District, Lanzhou 730030, China

*** Correspondence:**Corresponding Author: Wei Liang ([liangw@gsau.edu.cn](mailto:liangw@gsau.edu.cn)); Fengxia Guo (guofx@gsau.edu.cn); Yuan Chen (chenyuan@gsau.edu.cn).

**Table S1** Polysaccharide (POL), protein, ash, total polyphenol (TP), ethanol-soluble extract (ASE), water-soluble extract (WSE) contents.

| Samples | **POL (%)** | **Protein (%)** | **Ash (%)** | **TP (%)** | **ASE (%)** | **WSE (%)** |
| --- | --- | --- | --- | --- | --- | --- |
| FC | 10.56±0.51^Cc^ | 9.86±0.44^Aa^ | 3.08±0.02^Aa^ | 0.41±0.01^Aa^ | 75.16±0.48^Aa^ | 76.4±1.50^Aa^ |
| RD | 19.48±0.43^Aa^ | 7.61±0.16^Bb^ | 2.09±0.02^Bc^ | 0.38±0.02^Aab^ | 62.51±0.37^Bb^ | 65.68±0.82^Bb^ |
| SD | 15.3±0.25^Bb^ | 8.94±0.31^ABa^ | 2.91±0.06^Ab^ | 0.33±0.01^Ab^ | 59.59±0.71^Cc^ | 62.48±0.77^Bb^ |

**Note:** The data are presented as the means ± SD. POL, polysaccharide; TP, totalpolyphenol; ASE, ethanol-soluble extract; WSE, water-soluble extract; FC, Fresh *Codonopsis pilosula*; RD, "rubbing-sweating" dried *Codonopsis pilosula*; SD, Shade-dried *Codonopsis pilosula.* With in each column, the different superscripted small and capital letters Indicate significant and highly significant differences at *p*<0.05 and *p*<0.01, respectively, based on ANOVA-Duncan multiple comparison results.

**Table S2.** List of primers for RT-qPCR.

| No | Gene ID | KO name | Primer (5’-3’) |
| --- | --- | --- | --- |
| 1 | TRINITY_DN16881_c0_g1 | PR1 | F: GGTGAACGAGAGGCAGTACT  R: CGCGGGGAAAATAGTTGCAT |
| 2 | TRINITY_DN3379_c0_g1 | ETR | F: ACCTAACCCACGAGCTGAAA  R: GGCGTTCCACCTTTGAAGTT |
| 3 | TRINITY_DN3231_c1_g1 | GPX | F: TTTCACGCCCAATTGCTTCT  R: TCCCTTGTACTTGCTGAGCT |
| 4 | TRINITY_DN14343_c0_g1 | PDHB | F: TTCCCGTGGTTATTCGTGGA  R: CGGATCGCAGCTTTCATCAA |
| 5 | TRINITY_DN4979_c0_g1 | FAB2 | F: TGCCAAGGACTATGCGGATA  R: CGAATGTGCTCTCTCCTCCA |
| 6 | TRINITY_DN331_c1_g1 | SUS | F: CTAAGAACGCCAAGCTGAGG  R: TGCTGATAACCACCGGAACT |
| 7 | Actin | EF1α | F: GCCTGGTGACAACGTTGGAT  R: GCGAGGTGTGGCAATCAAGA |

**Table S3.** Some DEGs related to stress response and signal transduction in different dried *Codonopsis pilosula*.

| Gene No. | gene id | Description | KO id | KO name | Log _2_ (Fold Change) | | |  | | |  | | |
| --- | --- | --- | --- | --- | --- | --- | --- | --- | --- | --- | --- | --- | --- |
|  |  |  |  |  | RD vs SD | Significant | Regulate | RD vs FC | Significant | Regulate | SD vs FC | Significant | Regulate |
|  | **Peroxisome biogenesis** |  |  |  |  |  |  |  |  |  |  |  |  |
| 1 | TRINITY_DN9186_c0_g1 | peroxin-12 | K13345 | PEX12, PAF3 | 1.1137 | yes | up | 0.5432 | no | up | -0.5144 | no | down |
| 2 | TRINITY_DN4383_c1_g2 | peroxin-14 | K13343 | PEX14 | 1.0270 | yes | up | 0.1860 | no | up | -0.7866 | no | down |
| 3 | TRINITY_DN52325_c0_g1 | protein Mpv17 | K13348 | MPV17 | 2.1641 | yes | up | -0.0878 | no | down | -2.1871 | yes | down |
|  | **Amino acid metabolism** |  |  |  |  |  |  |  |  |  |  |  |  |
| 4 | TRINITY_DN3097_c0_g1 | superoxide dismutase | K04564 | SOD2 | 3.3733 | yes | up | 0.3198 | no | up | -2.9955 | yes | down |
| 5 | TRINITY_DN30358_c0_g3 | catalase | K03781 | CAT | -2.3767 | yes | up | -0.2926 | no | down | 2.1384 | yes | up |
| 6 | TRINITY_DN7989_c0_g1 | putative soluble epoxide hydrolase [Helianthus annuus]) | K08726 | EPHX2 | 4.6341 | yes | down | 1.0298 | no | up | -3.5526 | yes | down |
|  | Amino acid metabolism |  |  |  |  |  |  |  |  |  |  |  |  |
| 7 | TRINITY_DN21890_c0_g2 | PREDICTED: serine--glyoxylate aminotransferase [Ipomoea nil]) | K00830 | AGXT | 3.4373 | yes | up | -0.0306 | no | down | -3.4070 | yes | down |
| 8 | TRINITY_DN5693_c0_g1 | NADP-isocitrate dehydrogenase [Codonopsis lanceolata]) | K00031 | IDH1, | 1.2854 | yes | up | -1.1090 | yes | down | -2.3398 | yes | down |
| 9 | TRINITY_DN17771_c0_g2 | sarcosine oxidase / L-pipecolate oxidase | K00306 | PIPOX | 1.6775 | yes | up | 1.9156 | yes | up | 0.2900 | no | up |
| 10 | TRINITY_DN32428_c0_g2 | hydroxymethylglutaryl-CoA lyase, mitochondrial-like isoform X1 [Cynara cardunculus var. scolymus]) | K01640 | HMGCL | 1.0432 | yes | up | -0.4380 | no | down | -1.4249 | yes | down |
| 11 | TRINITY_DN17693_c0_g1 | FMN-dependent dehydrogenase [Corchorus capsularis]) | K11517 | HAO | 2.1209 | yes | up | -0.2728 | no | down | -2.3372 | yes | down |
|  | Glutathione metabolism |  |  |  |  |  |  |  |  |  |  |  |  |
| 12 | TRINITY_DN32316_c0_g2 | hypothetical protein VITISV_026403 [Vitis vinifera]) | K00383 | GSR | -1.5432 | yes | down | 1.6484 | no | up | 3.2506 | yes | up |
| 13 | TRINITY_DN5693_c0_g1 | NADP-isocitrate dehydrogenase [Codonopsis lanceolata]) | K00031 | IDH1, | 1.2854 | yes | up | -1.1090 | yes | down | -2.3398 | yes | down |
| 14 | TRINITY_DN6199_c0_g1 | 6-phosphogluconate dehydrogenase [Populus alba x Populus x berolinensis]) | K00033 | PGD | 1.0815 | yes | up | -0.6117 | no | down | -1.6385 | yes | down |
| 15 | TRINITY_DN53684_c0_g1 | glucose-6-phosphate 1-dehydrogenase | K00036 | G6PD | 1.5489 | yes | up | -1.1169 | yes | down | -2.6073 | yes | down |
| 16 | TRINITY_DN3231_c1_g1 | glutathione peroxidase | K00432 | GPX | -1.1397 | yes | down | 1.4322 | yes | up | 2.6211 | yes | up |
| 17 | TRINITY_DN8025_c0_g1 | ascorbate peroxidase | K00434 | E1.11.1.11 | 1.7265 | yes | up | 0.6870 | no | up | -0.9837 | no | down |
|  | a-Linolenic acid metabolism |  |  |  |  |  |  |  |  |  |  |  |  |
| 18 | TRINITY_DN12665_c0_g1 | coronatine insensitive 1, partial [Pyrus pyrifolia]) | K13463 | COI-1 | 1.1039 | yes | up | 0.5433 | no | up | -0.5062 | no | down |
| 19 | TRINITY_DN8404_c0_g1 | protein TIFY 6B isoform X1 [Populus trichocarpa]) | K13464 | JAZ | 1.8452 | no | up | -2.9434 | yes | down | -4.7441 | yes | down |
| 20 | TRINITY_DN14842_c0_g1 | transcription factor MYC2 | K13422 | MYC2 | 3.3189 | yes | up | -1.9536 | yes | down | -5.2184 | yes | down |
|  | Cysteine andme thiorine metabolism |  |  |  |  |  |  |  |  |  |  |  |  |
| 21 | TRINITY_DN26246_c0_g1 | ethylene receptor [Lonicera macranthoides]) | K14509 | ETR | 1.3210 | yes | up | 0.6988 | no | up | -0.5667 | no | down |
| 22 | TRINITY_DN1169_c4_g1 | serine/threonine-protein kinase CTR1-like isoform X2 [Vitis riparia]) | K14510 | CTR1 | 1.3191 | yes | up | -0.9851 | no | down | -2.2490 | yes | down |
|  | Phenylalanine mtabolism |  |  |  |  |  |  |  |  |  |  |  |  |
| 23 | TRINITY_DN884_c0_g1 | hypothetical protein F0562_029735 [Nyssa sinensis]) | K14508 | NPR1 | 1.6278 | yes | up | -0.2701 | no | down | -1.8427 | yes | down |
| 24 | TRINITY_DN16881_c0_g1 | pathogenesis-related protein 1 | K13449 | PR1 | 2.6841 | yes | up | 5.2790 | yes | up | 2.6509 | no | up |
|  | Pathogen infection |  |  |  |  |  |  |  |  |  |  |  |  |
| 25 | TRINITY_DN55749_c0_g1 | LRR receptor-like serine/threonine-protein kinase FLS2 | K13420 | FLS2 | 1.2360 | no | up | 3.1300 | yes | up | 1.9467 | no | up |
| 26 | TRINITY_DN1541_c0_g1 | brassinosteroid insensitive 1-associated receptor kinase 1 | K13416 | BAK1 | 1.7778 | yes | up | -1.3947 | yes | down | -3.1153 | yes | down |
| 27 | TRINITY_DN16881_c0_g1 | pathogenesis-related protein 1 | K13449 | PR1 | 2.6841 | yes | up | 5.2790 | yes | up | 2.6509 | no | up |
|  | Pathogen attack |  |  |  |  |  |  |  |  |  |  |  |  |
| 28 | TRINITY_DN2231_c1_g1 | hypothetical protein OSB04_027067 [Centaurea solstitialis]) | K20607 | MKK3 | 1.1277 | yes | up | -0.8886 | no | down | -1.9629 | yes | down |
| 29 | TRINITY_DN33060_c0_g1 | hypothetical protein L1987_08562 [Smallanthus sonchifolius]) | K20535 | MPK1 | 1.2714 | yes | up | -0.1582 | no | down | -1.3746 | yes | down |
| 30 | TRINITY_DN4223_c0_g1 | hypothetical protein F0562_003721 [Nyssa sinensis]) | K20537 | MPK7 | 2.0828 | yes | up | 0.1866 | no | up | -1.8419 | yes | down |
| 31 | TRINITY_DN3332_c0_g1 | nucleoside-diphosphate kinase | K00940 | NME | 1.6486 | yes | up | 0.4706 | no | up | -1.1223 | yes | down |
| 32 | TRINITY_DN7703_c0_g1 | mitogen-activated protein kinase kinase kinase ANP1 | K20606 | ANP1 | 1.4199 | yes | up | -1.0265 | yes | down | -2.3982 | yes | down |
|  | Drought/Osmotic stress |  |  |  |  |  |  |  |  |  |  |  |  |
| 33 | TRINITY_DN53837_c0_g1 | hypothetical protein FNV43_RR10175 [Rhamnella rubrinervis]) | K14496 | PYL | 1.2314 | no | up | -3.6323 | yes | down | -4.8007 | yes | down |
| 34 | TRINITY_DN15205_c1_g1 | serine/threonine-protein kinase SAPK7-like [Olea europaea var. sylvestris]) | K14498 | SNRK2 | 3.0046 | yes | up | 1.5745 | yes | up | -1.3748 | no | down |
| 35 | TRINITY_DN2231_c1_g1 | mitogen-activated protein kinase kinase 3 | K20607 | MKK3 | 1.1277 | yes | up | -0.8886 | no | down | -1.9629 | yes | down |
| 36 | TRINITY_DN33060_c0_g1 | mitogen-activated protein kinase 1 | K20535 | MPK1 | 1.2714 | yes | up | -0.1582 | no | down | -1.3746 | yes | down |
| 37 | TRINITY_DN4223_c0_g1 | mitogen-activated protein kinase 7 | K20537 | MPK7 | 2.0828 | yes | up | 0.1866 | no | up | -1.8419 | yes | down |
|  | **Ethylene** |  |  |  |  |  |  |  |  |  |  |  |  |
| 38 | TRINITY_DN28886_c0_g2 | transmembrane protein | K20726 | TMEM | 1.8907 | yes | up | -0.0246 | no | down | -1.8602 | yes | down |
| 39 | TRINITY_DN3379_c0_g1 | ethylene receptor | K14509 | ETR | 3.1428 | yes | up | -0.9825 | no | down | -4.0687 | yes | down |
| 40 | TRINITY_DN1169_c4_g1 | serine/threonine-protein kinase CTR1-like isoform X2 [Vitis riparia]) | K14510 | CTR1 | 1.3191 | yes | up | -0.9851 | no | down | -2.2490 | yes | down |
| 41 | TRINITY_DN13857_c0_g1 | endochitinase A [Nicotiana tomentosiformis]) | K20547 | CHIB | 1.3845 | yes | up | 1.4415 | yes | up | 0.1138 | no | up |
|  | Wounding responses |  |  |  |  |  |  |  |  |  |  |  |  |
| 42 | TRINITY_DN2231_c1_g1 | hypothetical protein OSB04_027067 [Centaurea solstitialis]) | K20607 | MKK3 | 1.1277 | yes | up | -0.8886 | no | down | -1.9629 | yes | down |
| 43 | TRINITY_DN14842_c0_g1 | hypothetical protein LWI28_015445 [Acer negundo]) | K13422 | MYC2 | 3.3189 | yes | up | -1.9536 | yes | down | -5.2184 | yes | down |
| 44 | TRINITY_DN14002_c0_g1 | calmodulin | K02183 | CALM | 4.4450 | yes | up | -0.5359 | no | down | -4.9291 | yes | down |
| 45 | TRINITY_DN2231_c1_g1 | hypothetical protein OSB04_027067 [Centaurea solstitialis]) | K20607 | MKK3 | 1.1277 | yes | up | -0.8886 | no | down | -1.9629 | yes | down |
| 46 | TRINITY_DN3770_c0_g1 | respiratory burst oxidase | K13447 | RBOH | 1.2276 | yes | up | -1.1491 | no | down | -2.3252 | yes | down |

**Table S4.** Some DEGs related to active ingredient synthesis in different dried *Codonopsis pilosula*.

| Gene No. | gene id | Description | KO id | KO name | Log _2_ (Fold Change) | | |  | | |  | | |
| --- | --- | --- | --- | --- | --- | --- | --- | --- | --- | --- | --- | --- | --- |
|  |  |  |  |  | RD vs SD | Significant | Regulate | RD vs FC | Significant | Regulate | SD vs FC | Significant | Regulate |
|  | **Phenylalanine, tyrosine and tryptophan biosynthesis** |  |  |  |  |  |  |  |  |  |  |  |  |
| 1 | TRINITY_DN46079_c0_g2 | phospho-2-dehydro-3-deoxyheptonate aldolase 2, chloroplastic [Lactuca sativa]) | K01626 | aroF | 1.5287 | yes | up | 2.3660 | yes | up | 0.8855 | no | up |
| 2 | TRINITY_DN27077_c0_g1 | 3-dehydroquinate synthase, chloroplastic [Helianthus annuus]) | K01735 | aroB | 1.3145 | yes | up | -0.8518 | no | down | -2.1130 | yes | down |
| 3 | TRINITY_DN19500_c0_g1 | bifunctional 3-dehydroquinate dehydratase/shikimate dehydrogenase, chloroplastic-like [Cynara cardunculus var. scolymus]) | K13832 | aroDE | 1.4710 | yes | up | -0.0159 | no | down | -1.4434 | yes | down |
| 4 | TRINITY_DN24447_c0_g1 | arogenate/prephenate dehydratase | K05359 | ADT | 1.9546 | yes | up | -0.2134 | no | down | -2.1135 | yes | down |
| 5 | TRINITY_DN7226_c1_g1 | histidinol-phosphate aminotransferase | K00817 | hisC | 1.4939 | yes | up | -1.1392 | yes | down | -2.5764 | yes | down |
| 6 | TRINITY_DN3107_c0_g3 | aspartate aminotransferase, chloroplastic isoform X2 [Cynara cardunculus var. scolymus]) | K00811 | GOT1 | 1.3650 | yes | up | 0.2698 | no | up | -1.0406 | yes | down |
| 7 | TRINITY_DN6501_c0_g1 | tyrosine aminotransferase-like isoform X1 [Cynara cardunculus var. scolymus]) | K00815 | TAT | 1.6338 | yes | up | -0.8833 | no | down | -2.4593 | yes | down |
| 8 | TRINITY_DN2966_c0_g1 | tryptophan synthase alpha chain | K06001 | trpB | 2.7747 | yes | up | -0.1960 | no | down | -2.9136 | yes | down |
| 9 | TRINITY_DN3679_c0_g2 | anthranilate synthase / indole-3-glycerol phosphate synthase | K01609 | trpC | 1.0478 | yes | up | -0.5756 | no | down | -1.5686 | yes | down |
| 10 | TRINITY_DN3999_c0_g2 | N-(5'-phosphoribosyl)anthranilate isomerase 1, chloroplastic-like isoform X1 [Camellia sinensis]) | K01817 | trpF | 2.1923 | yes | up | 0.0884 | no | up | -2.0480 | yes | down |
| 11 | TRINITY_DN3289_c0_g1 | anthranilate synthase beta subunit 1 [Prunus dulcis]) | K01658 | trpG | 2.6958 | yes | up | 0.8612 | no | up | -1.7774 | yes | down |
| 12 | TRINITY_DN10152_c0_g4 | anthranilate synthase alpha subunit 2, chloroplastic isoform X1 [Lactuca sativa]) | K01657 | trpE | 2.0728 | yes | up | -0.4518 | no | down | -2.4703 | yes | down |
|  | **Starch and sucrose metabolism** |  |  |  |  |  |  |  |  |  |  |  |  |
| 13 | TRINITY_DN3149_c1_g1 | beta-fructofuranosidase | K01193 | INV | 1.9079 | yes | up | 1.7070 | yes | up | -0.1440 | no | down |
| 14 | TRINITY_DN3917_c1_g1 | hexokinase [Actinidia deliciosa]) | K00844 | HK | -1.3176 | yes | down | 0.0270 | no | up | 1.3960 | yes | up |
| 15 | TRINITY_DN3855_c0_g1 | fructokinase-6 | K00847 | scrK | 1.0079 | yes | up | 0.2610 | no | up | -0.6927 | no | down |
| 16 | TRINITY_DN1021_c0_g2 | phosphoglucomutase, cytoplasmic-like [Juglans microcarpa x Juglans regia]) | K01835 | pgm | -1.3952 | yes | down | 1.6296 | yes | up | 3.0749 | yes | up |
| 17 | TRINITY_DN840_c2_g1 | UTP--glucose-1-phosphate uridylyltransferas | K00963 | UGP2 | 2.7275 | yes | up | -0.3681 | no | down | -3.0384 | yes | down |
| 18 | TRINITY_DN331_c1_g1 | sucrose synthase | K00695 | SUS | -1.9360 | yes | down | 3.8194 | yes | up | 5.8029 | yes | up |
|  | **Fatty acid biosynthesis/ Biosynthesis of unsaturated fatty acids** | |  |  |  |  |  |  |  |  |  |  |  |
| 19 | TRINITY_DN14343_c0_g1 | putative pyruvate dehydrogenase | K00162 | PDHB | -0.2815 | no | down | 1.3334 | yes | up | 1.6695 | yes | up |
| 20 | TRINITY_DN4247_c0_g1 | 3-oxoacyl-[acyl-carrier-protein] synthase II | K09458 | CEM1 | -3.2902 | yes | down | 1.9962 | yes | up | 5.3375 | yes | up |
| 21 | TRINITY_DN1024_c2_g1 | palmitoyl-acyl carrier protein thioesterase, chloroplastic-like [Cynara cardunculus var. scolymus]) | K10781 | FATB | -1.3647 | yes | down | 0.8343 | no | up | 2.2482 | yes | up |
| 22 | TRINITY_DN4979_c0_g1 | acyl-[acyl-carrier protein] desaturase | K03921 | FAB2 | -0.0352 | no | down | 1.2814 | yes | up | 1.3693 | yes | up |
| 23 | TRINITY_DN16071_c0_g1 | hypothetical protein Leryth_016841 [Lithospermum erythrorhizon]) | K10256 | FAD2 | 1.5269 | yes | up | 0.4182 | no | up | -1.0520 | yes | down |
|  | Amino sugar and nucleotide sugar metabolism |  |  |  |  |  |  |  |  |  |  |  |  |
| 24 | TRINITY_DN72_c0_g1 | UDP-glucose 4-epimerase | K01784 | galE | 1.3360 | yes | up | -3.4267 | yes | down | -4.7106 | yes | down |
| 25 | TRINITY_DN34612_c0_g1 | UDPglucose 6-dehydrogenase | K00012 | UGDH | 2.2598 | yes | up | -1.6050 | yes | down | -3.7992 | yes | down |
| 26 | TRINITY_DN5947_c0_g1 | UDP-apiose/xylose synthase | K12449 | AXS | 1.2932 | yes | up | 0.7990 | no | up | -0.4394 | no | down |
| 27 | TRINITY_DN4554_c0_g1 | UDP-arabinopyranose mutase | K13379 | RGP | 1.4681 | yes | up | 1.8706 | yes | up | 0.4594 | no | up |
| 28 | TRINITY_DN1113_c0_g1 | UDP-glucose 4,6-dehydratase | K12450 | RHM | 1.2019 | yes | up | 1.4983 | yes | up | 0.3519 | no | up |
| 29 | TRINITY_DN1574_c0_g1 | mannose-1-phosphate guanylyltransferase | K00966 | GMPP | 1.6782 | yes | up | -0.1112 | no | down | -1.7341 | yes | down |
